# Advances and Challenges in Immune-Modulatory Biomaterials for Wound Healing Applications

**DOI:** 10.3390/pharmaceutics16080990

**Published:** 2024-07-26

**Authors:** Yuqi Cao, Jiagui Sun, Shengao Qin, Zhengshu Zhou, Yanan Xu, Chenggang Liu

**Affiliations:** 1 School Basic Medical Sciences, Heilongjiang University of Chinese Medicine, 24 Heping Road, Harbin 150040, China; qq1656445881@163.com (Y.C.); 13209846730@139.com (J.S.); zhzsyydsh@163.com (Z.Z.); 15838399049@163.com (Y.X.); 2 Beijing Laboratory of Oral Health, Capital Medical University, 10 Xitoutiao, Beijing 100054, China; shengaoqin123@163.com

**Keywords:** skin wound healing, biomaterials, mitochondria, immune regulation, plant-derived drugs

## Abstract

Wound healing progresses through three distinct stages: inflammation, proliferation, and remodeling. Immune regulation is a central component throughout, crucial for orchestrating inflammatory responses, facilitating tissue repair, and restraining scar tissue formation. Elements such as mitochondria, reactive oxygen species (ROS), macrophages, autophagy, ferroptosis, and cytokines collaboratively shape immune regulation in this healing process. Skin wound dressings, recognized for their ability to augment biomaterials’ immunomodulatory characteristics via antimicrobial, antioxidative, pro- or anti-inflammatory, and tissue-regenerative capacities, have garnered heightened attention. Notwithstanding, a lack of comprehensive research addressing how these dressings attain immunomodulatory properties and the mechanisms thereof persists. Hence, this paper pioneers a systematic review of biomaterials, emphasizing immune regulation and their underlying immunological mechanisms. It begins by highlighting the importance of immune regulation in wound healing and the peculiarities and obstacles faced in skin injury recovery. This segment explores the impact of wound metabolism, infections, systemic illnesses, and local immobilization on the immune response during healing. Subsequently, the review examines a spectrum of biomaterials utilized in skin wound therapy, including hydrogels, aerogels, electrospun nanofiber membranes, collagen scaffolds, microneedles, sponges, and 3D-printed constructs. It elaborates on the immunomodulatory approaches employed by these materials, focusing on mitochondrial and ROS modulation, autophagic processes, ferroptosis, macrophage modulation, and the influence of cytokines on wound healing. Acknowledging the challenge of antibiotic resistance, the paper also summarizes promising plant-based alternatives for biomaterial integration, including curcumin. In its concluding sections, the review charts recent advancements and prospects in biomaterials that accelerate skin wound healing via immune modulation. This includes exploring mitochondrial transplantation materials, biomaterial morphology optimization, metal ion incorporation, electrostimulation-enabled immune response control, and the benefits of composite materials in immune-regulatory wound dressings. The ultimate objective is to establish a theoretical foundation and guide future investigations in the realm of skin wound healing and related materials science disciplines.

## 1. Introduction

The skin, constituting the body’s largest organ and primary defense mechanism, is susceptible to irritation and damage due to prolonged exposure. Consequently, various types of skin wounds can emerge, broadly classified into acute and chronic categories [1]. The prevalence of wounds is substantial, leading to significant treatment expenses. By 2021, the global wound care market had exceeded $20.59 billion. In the United States alone, over 17.2 million acute wounds were diagnosed in 2014. Additionally, chronic wounds afflict approximately 1–2% of the population in developed nations. Overall, it is estimated that more than 1 billion individuals worldwide contend with both acute and chronic wounds [2,3]. Wounds represent a significant healthcare concern on a global scale, demanding attention and intervention. The presence of skin wounds can disrupt a patient’s daily activities, and inadequate wound management increases the risk of infection, potentially leading to severe consequences such as amputation or mortality [4]. Skin wound healing comprises three distinct phases: the inflammatory phase, which includes the hemostatic phase; the proliferative phase; and the regenerative phase [5]. Immunomodulatory responses play a pivotal role throughout the entire wound healing process, crucially regulating the inflammatory response, facilitating repair, and controlling scar formation. Any factor influencing wound healing impacts both the rate and quality of the process, with an aberrant inflammatory response identified as the primary culprit [6]. An insufficient inflammatory response compromises the efficacy of immune cells, such as macrophages, against microorganisms, potentially leading to wound infection and impeding early wound regeneration. Conversely, an excessive or prolonged inflammatory response may hinder the progression from the inflammatory phase to the proliferative phase, significantly impeding wound regeneration and predisposing the wound to scarring or chronic non-healing [7,8]. Wound infections further exacerbate inflammation, thereby hindering wound healing. Continuous research and development efforts are underway to devise various types of wound dressings aimed at preventing infections and expediting wound healing.

Immunomodulation in skin wound healing is primarily influenced by macrophages. Macrophages are pivotal regulators throughout the entire wound healing process. Their behavior, phenotype, and polarization significantly impact both wound inflammation and regeneration. Mitochondria, ROS, and cellular autophagy also influence immunomodulation and macrophage polarization, thereby affecting both inflammation and regeneration processes. Furthermore, inducing iron-mediated cell death in bacteria within wounds demonstrates potent antimicrobial effects without eliciting drug resistance or adverse reactions. Therefore, the modulation of these factors is essential to regulate the immune response and promote wound healing.

The limitations of conventional wound dressings, encompassing both their physical properties and therapeutic efficacy, pose significant challenges to effective wound healing. Commonly used traditional dressings, such as gauze and cotton, suffer from suboptimal adhesive qualities, fixation difficulties, incomplete coverage, and inadequate antimicrobial properties, necessitating frequent changes. Moreover, these dressings are prone to accumulating exudate or blood, leading to their adherence to wound tissues, which can result in secondary hemorrhages and discomfort during dressing changes [9,10,11]. Consequently, various novel wound dressings have been developed to address these deficiencies. Additionally, there has been a growing focus on wound dressings that facilitate healing through immunomodulation, demonstrating significant advancements in drug delivery and immunomodulatory capabilities. Despite intensive study of several immunomodulatory biomaterials, there remains a notable absence of systematic reviews on wound dressings with immunomodulatory properties, as well as a lack of comprehensive research on the acquisition of these abilities and their underlying mechanisms. This study aims to fill these gaps by introducing the critical role of immune regulation in the wound healing process and systematically reviewing biomaterials targeting immune modulation and their mechanisms. This will provide a foundational understanding and guidance for future research in wound healing and related materials science fields.

This paper underscores the critical role of wound metabolism, infection, systemic diseases exacerbating wound injuries, and local impediments in skin injury and repair. We then explore various biomaterials, including hydrogels, aerogels, collagen scaffolds, electrostatically spun nanofiber membranes, microneedles, sponges, and 3D-printed materials, which have shown effectiveness in enhancing wound healing through immunomodulation. Specifically, we discuss the roles of mitochondria, ROS, autophagy, iron-induced cell death, macrophage polarization, and cytokines in wound healing immunomodulation. Additionally, we investigate the potential of loading biomaterials with plant extracts to enhance wound healing by modulating the immune microenvironment, thereby addressing concerns related to drug resistance and adverse reactions and aiming to supplant traditional antibiotics. In conclusion, we highlight recent advancements and future prospects in biomaterials for wound healing via immunomodulation, with a focus on emerging biomaterials and their less-explored immunomodulatory mechanisms such as mitochondrial grafts, biomaterial morphology, metal ions, and composites. These insights present promising avenues for future research in wound healing, offering valuable direction and inspiration for further study in this field.

## 2. Skin Injuries and Repair Difficulties

**Figure 1:**
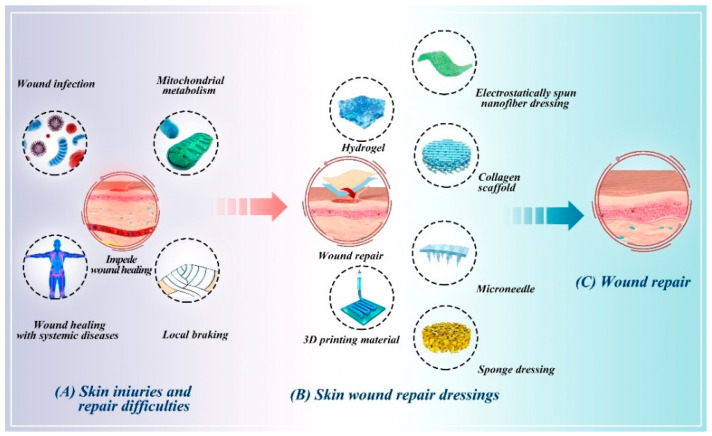
Skin injuries, repair difficulties, and several types of wound dressings with immunomodulatory functions.

The healing of skin injuries is primarily influenced by wound metabolism, infections, injuries associated with systemic diseases, and local impediments. These factors significantly impact the speed and quality of wound healing by influencing the immune response (Figure 1A).

### 2.1. Influence of Wound Metabolism on the Immune Response

Metabolism is the collective term for the chemical reactions that sustain the life processes of an organism, which can be categorized into material metabolism and energy metabolism, including catabolism and anabolism [12]. Proper regulation is crucial for wound healing, as it can effectively modulate the immune response.

On the one hand, local immunomodulation is influenced by energy metabolism, including mitochondrial metabolism, glycolysis, and lipid metabolism. Specifically, mitochondria produce ATP and metabolic precursors via oxidative phosphorylation (OxPhos) to supply energy for wound healing, along with ROS that regulate a suitable pro-inflammatory and vascular response in the early stages of wound closure. Simultaneously, mitochondria serve as metabolic regulation hubs essential for activating various types of immune cells in both the innate and acquired immune systems. Mitochondrial metabolism induces apoptosis in platelets and certain immune cells, while mitochondrial autophagy contributes to macrophage metabolic reprogramming, influencing M1 or M2 macrophage functions. Secondly, glycolysis serves as a primary energy source in the initial stages of wound healing, preventing excessive ROS production and rapidly replenishing energy. The upregulated glycolysis supports the pro-inflammatory and early pro-angiogenic effects of M1 macrophages during the inflammatory phase. Additionally, glycolysis produces essential intermediates for biosynthesis, sustaining macrophage and neutrophil function in the inflammatory phase and promoting tissue regeneration through supporting anabolic processes [13,14,15]. Lipid signaling mediates cellular processes and intercellular communication during skin wound healing and tissue regeneration [12]. While specific lipids are necessary to maintain the inflammatory response during the inflammatory phase, free fatty acids’ lipids remain chronically upregulated even after the normal course of wound healing, potentially prolonging wound inflammation and maintaining wound healing in the inflammatory phase [14].

On the other hand, diverse macrophage metabolic activities during the early and late phases of wound healing profoundly influence the immune response. Cellular metabolism intricately links to immune cell activation and function, defining distinct macrophage phenotypes in early and late-stage wounds. Initially, wound macrophages exhibit an M1 phenotype characterized by predominant anabolic metabolism. As the wound progresses, macrophages transition from M1-like/glycolytic to M2-like/oxidative phenotypes, with late-stage M2 macrophages predominantly displaying catabolic metabolism. M1 macrophages primarily orchestrate the inflammatory phase, relying on glycolysis and heightened mitochondrial activity (e.g., mitochondrial ROS production) to evoke early antimicrobial, pro-inflammatory, and vascular responses crucial for timely wound healing. In contrast, M2 macrophages become prominent in later stages of wound healing, favoring oxidative phosphorylation (OxPhos) and exhibiting increased catabolic activity akin to an immune-tolerant or dormant state. This supports tissue protection and homeostasis during the reparative process. Perturbations in metabolic reprogramming can disrupt the proper transition of macrophage subtypes, thereby impacting wound healing outcomes [13,14].

### 2.2. Wound Infection

Any compromise during wound healing can disrupt the wound environment and impede the healing process. Among these, wound infection stands as the most prevalent and avoidable obstacle, often culminating in treatment failure and non-healing [16]. Infections commonly manifest during the inflammatory phase, characterized by wound exudate, persistent inflammation, and severe pain [17]. Pathogenic invasion and proliferation, notably by β-hemolytic streptococci, *Staphylococcus aureus*, and *Pseudomonas aeruginosa*, directly trigger wound infections [18,19]. Microbial contamination triggers an extensive inflammatory response, leading to macrophage polarization failure, heightened pro-inflammatory cytokine production, and the overexpression of matrix metalloproteinases (MMPs), which degrade the extracellular matrix (ECM). Consequently, granulation tissue, neovascularization, and re-epithelialization are inhibited, resulting in delayed and impaired wound repair [17,20,21,22]. If the infection persists, the wound becomes chronic. A complex community of polymicrobial biofilms is common on the surface of infected chronic wounds [23]. The persistent inflammation caused by biofilms leads to excessive and persistent NETosis, while the formation and accumulation of dead tissue and exudate increases biofilm production, creating a vicious cycle [22]. Persistently open wounds remain vulnerable to continual microbial influx, exacerbating infection and complicating healing [24]. Moreover, biofilms not only compromise the host immune response but also hinder epithelial cell tight junctions, impeding wound closure and fostering antibiotic resistance. This poses significant challenges for both autoimmunity and external treatments [23,25].

Consequently, preventing wound infection not only mitigates ongoing inflammation and tissue damage but also facilitates prompt wound healing. Various antimicrobial dressings with immunomodulatory properties have emerged, capable of isolating pathogenic bacteria from the wound environment and acting as bactericidal, anti-inflammatory, and tissue-regenerative agents through drug loading or synergistic approaches. Common strategies include incorporating antimicrobial materials like dopamine-modified hyaluronic acid and integrating artificial antibiotics, natural plant extracts, bioactives, and metal ions into dressings to confer antimicrobial effects. Examples include thymol, glycyrrhizin, doxycycline, antimicrobial peptides, bionic neutrophil nanoparticles, and silver nanoparticles (AgNPs) [26,27,28,29,30,31]. Dressings with electrical stimulation capabilities represent an emerging antimicrobial strategy, inducing oxidative stress in bacteria through increased intracellular ROS, thereby damaging bacterial membranes and exhibiting broad-spectrum antimicrobial effects against Gram-positive/negative bacteria [32]. Simultaneously, electrical stimulation fosters tissue regeneration and wound healing, offering a drug-resistance-free and biologically safe physical stimulation method [33,34]. Si et al. developed an electrically conductive organic hydrogel with antimicrobial and antioxidant properties, promoting wound healing by reducing inflammatory responses and facilitating angiogenesis [35].

Moreover, while various approaches exist to impart antimicrobial properties to dressings, conventional dressings often struggle to effectively inhibit bacterial membranes. In contrast, microneedle dressings uniquely penetrate biofilms, effectively inhibiting their growth while preserving their immunomodulatory functions [36]. Yang et al. developed microneedles loaded with AgNPs, designed to penetrate the physical barrier of biofilms and exert antimicrobial efficacy. These microneedles also promote wound healing through antioxidant mechanisms and the suppression of excessive inflammation [37]. Additionally, Ouyang et al. developed microneedles loaded with the NLRP3 inhibitor MCC950, which enhances wound healing by curtailing inflammation triggered by NLRP3 inflammasome activation due to bacterial infection [38].

### 2.3. Wound Healing with Systemic Diseases

Systemic diseases pose significant challenges to wound healing by disrupting immune regulation, with diabetes mellitus being one of the most prevalent conditions. Chronic diabetic cutaneous ulcers, particularly in the lower extremities, are common complications known as diabetic foot ulcers [39]. Pathophysiological factors contributing to impaired diabetic wound healing include hyperglycemia, infections, neuropathy, immunodeficiency, and aberrant ECM degradation from previous diseases. Elevated levels of hyperglycemia compromise immune cell function, diminish the anti-inflammatory stromal cell protein ANGPTL4, impede the leukocyte migration crucial for infection control, and promote the overexpression of serum pro-inflammatory cytokines such as interleukins (ILs), Tumor Necrosis Factor-α (TNF-α), monocyte chemoattractant protein-1 (MCP-1), and MMPs [40,41,42,43]. Moreover, hyperglycemia sustains inflammatory macrophages at the wound site, inhibiting their polarization towards an anti-inflammatory phenotype. This leads to excessive ROS and pro-inflammatory cytokine production, prolonging inflammation. It also induces abnormal fibroblast apoptosis and impairs keratinocyte and angiogenic responses, ultimately hindering angiogenesis and granulation tissue formation, thus delaying wound healing [44,45]. Diabetic patients are more susceptible to chronic wounds due to compromised immune function and increased microbial susceptibility resulting from elevated blood glucose levels. Additionally, diabetic wounds show enhanced recruitment of pro-inflammatory cytokines by myeloid cells and the release of pro-inflammatory cytokines due to extravascular advanced glycation end products and neutrophil binding, exacerbating the inflammatory response [40,43]. Furthermore, wound hypoxia, iron death, and ferritin phagocytosis resulting from hyperglycemia further impede wound healing by disrupting cellular function, angiogenesis, and tissue regeneration [46,47].

Other prevalent systemic diseases, such as human immunodeficiency virus (HIV) with acquired immunodeficiency syndrome (AIDS) and renal failure, pose significant challenges to wound healing due to immune dysfunction. Patients with HIV/AIDS experience compromised immune systems, making them highly susceptible to wound infections, particularly fungal infections of the skin, which can impede healing by spreading through the wound. HIV further delays wound healing by reducing CD4+ levels in immune cells. Additionally, many non-AIDS HIV patients exhibit comorbid metabolic syndromes like diabetes mellitus, further increasing their risk of wound healing complications [48,49]. Patients undergoing continuous renal replacement therapy (CRRT) for renal failure are also prone to difficulties in wound healing due to their being immunocompromised. CRRT induces the loss of trace elements such as Cu^2+^ and Zn^2+^, which are crucial for wound healing and immune defense. Continuous venous hemodiafiltration disrupts copper and zinc metabolism, compromising patient immunity and impacting wound healing [50].

In recent years, there has been a significant focus on developing diabetic wound dressings with immunomodulatory properties. Some dressings not only promote wound healing but also lower blood glucose levels, enhancing their anti-inflammatory and pro-regenerative efficacy. These advancements have resulted in more effective healing of diabetic wounds. However, there is still a noticeable lack of wound healing dressings designed to meet the challenges faced by individuals with other systemic diseases that often complicate wound healing. Future research efforts should prioritize developing such dressings to address the unmet needs in this area.

### 2.4. Local Braking

Movement-related challenges significantly impede wound healing in specific anatomical sites. Wound dressings intended for these areas are susceptible to displacement or damage, increasing the risk of infection and hindering healing. These issues are particularly prevalent in joints, muscle folds, and soles, which undergo frequent flexion during daily activities. This not only delays wound healing but also compromises the efficacy of the dressings [10,51,52]. Excessive or insufficient adhesion, along with the inadequate tensile strength and flexibility of dressings, can cause discomfort for patients, increase the likelihood of dressing damage or detachment, elevate the risk of infection, and potentially lead to secondary injuries [53].

Most wound dressings are primarily designed for flat skin wounds, with limited attention given to specialized areas [54]. Research on wound dressings for these regions primarily focuses on hydrogels, with limited exploration into film and 3D-printed dressings. For instance, Wang et al. developed a flexible bandage-based asymmetric wound dressing consisting of a composite hydrogel incorporating Melatonin and polyacrylamide. This dressing demonstrates excellent skin adhesion and deformation capability, accommodating joint movements effectively [52]. Hu et al. developed a sprayable zwitterionic antibacterial hydrogel with favorable adhesion and flexibility for application on joints, promoting angiogenesis and reducing inflammation [55]. Shan et al. introduced a composite hydrogel incorporating hydroxylated graphene, polysaccharide biopolymer, and poly(vinyl alcohol) (PVA), offering antimicrobial properties and preventing re-tearing while monitoring joint wound conditions [56]. Zhang et al. designed an antimicrobial hydrogel composed of iron(III) chloride, PVA, tannic acid, and borax, exhibiting multifunctional antimicrobial properties with self-healing, stretching, and shape adaptation capabilities for joint application [57]. Nanofiber films, known for their high stretchability, breathability, and rapid drug release, are suitable for joint wounds [54]. Lee et al. developed a Janus-structured polydimethylsiloxane film, stretchable to up to 150% of its original length, suitable for joint wounds [58]. Zhou et al. fabricated a bionic conductive dressing utilizing water-induced self-assembly and electrostatic spinning technology, aligning wrinkles and monitoring joint wound conditions [59]. In addition, certain new 3D printing materials demonstrate high flexibility and resistance properties, making them suitable for specialized wounds. Hu et al. introduced a biocompatible and highly flexible DLP resin for 3D printing, capable of creating complex dressings for finger joints. These dressings promote wound healing by modulating inflammation, granulation, and angiogenesis [60]. Zhong et al. developed a Gel-dECM-Qcs composite scaffold with antimicrobial properties through 3D printing technology, featuring good tensile and flexural resistance for diabetic foot wounds [61]. However, the limited number of studies on these materials necessitates subsequent verification for large-scale clinical use.

## 3. Several Types of Wound Dressings with Immunomodulatory Functions

Immunomodulation plays a crucial role in wound healing, and dressings that facilitate this process have gained significant attention. In recent years, numerous wound dressings with immunomodulatory properties have been developed and shown to enhance wound healing efficacy. The following discussion outlines several prevalent types of wound dressings, which can be tailored for immunomodulation through various strategies. Among these, hydrogels and electrostatically spun nanofibrous membranes are particularly prominent approaches (Figure 1B,C).

### 3.1. Hydrogels

A hydrogel is a polymer material characterized by its three-dimensional network structure [62]. A hydrogel as a wound dressing has outstanding advantages, mainly including the following three points: (1) A hydrogel exhibits excellent physical properties, including a soft structure, superior tensile strength, high porosity, elevated water content, and exceptional oxygen permeability. (2) A hydrogel exhibits good biocompatibility, resembling the ECM and displaying heightened sensitivity to the physiological environment. It effectively maintains wound moisture, alleviates pain, promotes new cell growth, facilitates hemostasis and wound secretion absorption, and ensures easy and painless removal, all while being biodegradable. (3) Acting as a substance delivery platform, a hydrogel serves as an effective carrier for a wide range of substances, such as drugs, exosomes, small molecules, stem cells, proteins/peptides, mitochondria, and nanoparticles. These substances can be encapsulated within the hydrogel matrix or coupled to the polymer chain for drug delivery, effectively preventing drug degradation and controlling release rates [63,64,65,66,67,68].

Various strategies can enhance the efficacy of hydrogel dressings in promoting wound healing through immunomodulation. Hydrogels can be engineered to exhibit antimicrobial and anti-inflammatory properties, which attenuate inflammation by modulating macrophages and immune cytokines. Common strategies include optimizing the preparation process, using specialized materials, designing unique structures, and incorporating bioactive ingredients such as probiotics, protease inhibitors, antimicrobial peptides, bionic vesicles, Haematococcus, stem cells, and anti-inflammatory cytokines. Additionally, loading botanical extracts, drugs, antibiotics, metal ions, and incorporating electrical stimulation are recognized approaches [69,70,71,72,73,74,75,76,77,78,79,80,81,82].

### 3.2. Aerogel

An aerogel is an ultra-lightweight solid material with 99% air, and its various outstanding properties and characteristics make it very suitable for use as a wound dressing [83]. Firstly, aerogels exhibit low density, light weight, excellent air permeability, high porosity, a substantial specific surface area, and a multistage porous network. Second, aerogels possess significant biomedical relevance, as they can absorb wound exudates, regulate wound moisture and pH balance, maintain physiological temperature, promote cell attachment, reduce inflammation, and prevent bacterial infections. Thirdly, as a drug delivery platform, aerogels can store drugs, enhance drug stability, facilitate drug release, improve the bioavailability of insoluble drugs, and mitigate drug toxicity. Fourthly, aerogels can be easily modified by adjusting pore size, thereby influencing various effects such as cell adhesion, permeability, oxygen permeability, fluid absorption, metabolite exchange, and drug release rates [84,85,86,87]. Furthermore, by loading metal ions and plant extracts, aerogels can be engineered to promote wound healing through immunomodulation [88,89].

A hydrogel absorbs wound exudate more slowly than an aerogel, which may lead to disadvantages such as inconvenient handling and difficulty in sterilization, and can increase the risk of wounds becoming infected, which can be circumvented by aerogels [90]. However, aerogels may sometimes irritate the skin of some individuals, leading to dryness of skin [83]. Hydrogel–aerogel biphasic gels can combine the advantages and mitigate the disadvantages of both components, maintaining efficacy in promoting wound healing through immunomodulation. Zou et al. developed a hydrogel–aerogel biphasic gel comprising bio-based polyurethane and PAMs-grafted PVA. This gel exhibits rapid exudate absorption similar to aerogels and liquid absorption and retention akin to hydrogels. Importantly, it possesses immunomodulatory abilities that promote macrophage polarization towards the M2 type, thereby reducing inflammatory responses. Simultaneously, it enhances collagen deposition and vascular regeneration to facilitate wound healing [91].

### 3.3. Electrostatically Spun Nanofiber Dressings

Nanofibers are fibers with nanoscale dimensions, which are very conducive to wound healing, and their advantages include having high similarity to the ECM, which promotes the secretion and remodeling of the ECM as well as cell attachment, migration, proliferation, and differentiation; having inert cell-like properties that make it painless to remove when used as a wound dressing and for the protection of the newly formed skin tissues while avoiding the formation of scars, etc., which are commonly used to make wound healing dressings [92,93]. The main techniques for manufacturing nanofiber dressings are electrostatic spinning, solution blow spinning, thermally induced phase separation, self-assembly, template synthesis, etc., of which electrostatic spinning is the most commonly used [94,95]. Most nanofiber dressings are prepared using electrostatic spinning technology due to its ease of fabrication, the convenient control of fiber diameter, the high polymer feasibility, and the fact that electrostatically spun nanofibers are 18–21% more absorbent than films fabricated using other techniques from the same raw material [93,95,96].

Electrostatically spun nanofiber dressings offer several advantages. First, they exhibit good adhesion, a high specific surface area, and high porosity, enabling excellent breathability and water absorption, which helps in absorbing wound secretions and preventing wound dehydration. Second, their porous three-dimensional structure provides robust mechanical strength. Third, nanofibers have a strong encapsulation ability and their porous structure makes them highly suitable for loading various drugs. Special structures such as multilayer, core–shell, and Janus structures can be tailored to control the solubility and release rates of drugs, facilitating multiple, burst, and sustained releases. Fourth, their structural similarity to the ECM results in minimal immune rejection. Fifth, the size, morphology, porosity, and mechanical properties of electrostatically spun nanofiber dressings can be adjusted to create appropriately sized wound dressings for wounds of any size [93,95,97,98,99]. Compared to other types of dressings, the unique advantage of electrostatically spun nanofibrous membranes lies in their surface roughness and porous morphology characteristics, which promote cell attachment, adhesion, migration, proliferation, and angiogenesis [100].

A number of strategies can be used to make electrostatically spun nanofibrous membrane dressings with immunomodulatory capabilities to attenuate the inflammatory response by modulating macrophage polarization and cytokine secretion, as well as antimicrobial and wound regenerative capabilities to promote wound healing. Common approaches include the following: the use of specific materials, the loading of synthetic compounds, the loading of plant extracts, and the loading of growth factors or metal ions [101,102,103,104,105].

### 3.4. Collagen Scaffold

Collagen scaffolds are composed of collagen that mimics the ECM and has the advantages of low irritation, low cytotoxicity, non-immunogenicity, high biocompatibility, hypoallergenicity, biodegradability, and superior bioactivity. The unique advantage of collagen scaffolds for wound healing compared to other dressings is that they provide excess collagen for destruction by MMPs, thus preventing the destruction of the healthy and nascent ECM and slowing wound healing [106,107]. Collagen scaffolds exhibit good structural integrity, breathability, and provide collagen fibers for ECM rebuilding, while also serving as a biochemical scaffold for cell growth, macromolecular attachment, and anchoring. Loading collagen scaffolds with metal ions, natural compounds, and living cells (e.g., mesenchymal stem cells) can enable them to promote wound healing through immunomodulation [108,109,110,111,112].

However, collagen scaffolds also present certain limitations that restrict their application, such as rapid degradation, poor mechanical properties, water resistance, and thermal stability [110]. Addressing these limitations, the use of natural, non-toxic, and biodegradable polysaccharides as bio-crosslinking agents emerges as a promising strategy. This approach offers a solution to the challenges posed by wound dressings without compromising their immunomodulatory capabilities. For instance, Gopika Selvakumar et al. utilized oxidized pullulan as a bio-crosslinking agent to fabricate a novel collagen scaffold matrix. Through this method, they developed a collagen scaffold loaded with p-coumaric acid, effectively reducing the inflammatory response and shortening the inflammatory period by mitigating inflammatory cells, while promoting collagen deposition and wound healing. The study demonstrated that oxidized branched starch enhanced the mechanical properties and stability of the collagen scaffold without compromising its biocompatibility or immunomodulatory capacity [113].

### 3.5. Microneedle

Microneedles represent a novel form of transdermal wound dressing that administers drugs via micron-sized needles. Compared to traditional wound dressings, microneedles offer unique advantages: their penetration can disrupt bacterial membranes, scabs, or scars. By penetrating the skin and stratum corneum, they increase the contact area for drug delivery directly to specific cortical layers. This method enables the painless administration of macromolecules, bypasses first-pass metabolism, and avoids damage to skin nerve endings. Moreover, microneedles provide more accurate test results compared to other wound dressings used for testing, as they directly contact the wound. Additionally, the mechanical stimulation induced by the needle array structure alters the local stress environment, promoting collagen deposition, tissue regeneration, and accelerated wound healing. Microneedles can also load multiple drugs simultaneously, offering high delivery efficiency, adjustable release rates, and enhanced utilization [6,114,115].

By integrating electrical stimulation therapy and loading plant extracts, synthetic compounds, metal ions, and bioactive substances (such as proteins or exosomes), microneedle dressings can acquire immunomodulatory capabilities to reduce inflammation, scavenge ROS, exhibit antimicrobial properties, promote tissue regeneration, and facilitate wound healing by modulating macrophage or cytokine levels [6,27,38,116,117,118,119].

However, the vertical needle body of microneedle dressings poses challenges, as it results in a weak grip, reducing adhesion and drug penetration. Utilizing bionics in microneedle manufacturing can address these issues without compromising immunomodulatory efficacy [6]. Liu et al. developed a porcupine quill-like bionic microneedle with a hydrogel-backed patch, which exhibited enhanced tissue adhesion strength and mechanical properties by mimicking the barbs of the African porcupine quill. Additionally, this dressing demonstrated the ability to inhibit wound inflammation and exhibit antimicrobial activity, thus promoting diabetic wound healing [120].

### 3.6. Sponge Dressings

A sponge material is a three-dimensional porous material characterized by having low density, a high specific surface area, high porosity, tissue compatibility, and appropriate mechanical properties. Sponge dressings excel in breathability, the absorption of wound exudate, the maintenance of wound wetness, sustained drug release, and the stabilization of the wound microenvironment [121,122,123]. Among their various features, sponge dressings are particularly renowned for their hemostatic ability. Their unique coded structure enables the rapid recruitment of blood cells and coagulation factors, facilitating hemostasis. Additionally, shape memory sponges can address deep, incompressible bleeding in deeper wounds [122,124]. Sponge dressings can be rendered immunomodulatory through the physical or chemical modification of the material, loading with living cells or exosomes, the incorporation of metal ions, and the inclusion of plant extracts to promote wound healing through antibacterial, anti-inflammatory, and tissue regeneration mechanisms [121,125,126,127,128].

However, sponge dressing adhesion is typically weak, which can affect the release and efficacy of their loaded components and their ability to absorb wound exudate. A promising strategy to address this issue without compromising immunomodulatory abilities involves a combination of biomimetic and enzymatic cross-linking reactions. Li et al. developed sponge dressing matrices by cross-linking gelatin and dopamine-modified hyaluronic acid. They then loaded titanium dioxide nanoparticles and polyhexamethylene biguanide onto the sponge matrix to create a multifunctional hybrid sponge dressing. This dressing exhibited good wet adhesion, mechanical properties, and biocompatibility. Furthermore, it reduced inflammatory responses by modulating the expression of inflammatory factors, while promoting the healing of infected burn wounds through antimicrobial activity, the scavenging of ROS, and the promotion of tissue regeneration [129].

### 3.7. Three-Dimensional Printing Materials

Three-dimensional bioprinting is a rapid prototyping technology based on on-demand-designed three-dimensional models, positioning, and assembling biocompatible materials for layer-by-layer deposition. Common wound dressings can be combined with 3D printing technology to produce dressings that better suit people’s needs [130]. Three-dimensional printing materials can mimic complex cellular tissue structures and the ECM to produce dressings containing a variety of biologically active molecules and cellular components in structures similar to natural tissues. Because 3D printing materials are constructed layer by layer according to a predetermined computer model, 3D printing technology holds excellent potential for modulating immune responses to promote wound healing by tailoring the structure, pore size, and morphology of the dressings. However, 3D-printed materials face numerous challenges, including a lack of suitable inks for printing, difficulties in nutritional supply, and reduced mechanical strength over time [94,130,131].

In recent years, researchers have developed bioinks with excellent properties for promoting wound healing, along with immunomodulatory capabilities, which have addressed these issues to some extent. For instance, Francesco Patitucci et al. developed an alginate/pectin-based bioink, enabling the preparation of a dressing with high biocompatibility and excellent anti-inflammatory and antioxidant properties, promoting cell motility and wound healing, as well as absorbing wound exudates [132]. Similarly, Li et al. created a 3D-printed bioink using sodium alginate and M2 macrophage-derived extracellular vesicles. This ink could modulate wound inflammation, promote macrophage polarization to the M2 type, enhance the proliferation and migration of vascular endothelial cells, induce angiogenesis, and facilitate wound healing. Additionally, the 3D printing technology enabled the ink to adapt to wounds of arbitrary sizes and shapes [133].

However, several challenges remain unresolved in 3D printing technology. These include optimizing printing speed and resolution levels, achieving structures that effectively stimulate the skin, and accurately mimicking the layered structure of skin tissues. Additionally, the naming systems and protocols for different printing technologies vary widely, complicating the widespread adoption and standardization of 3D printing in wound care [130].

## 4. Immunomodulatory Strategies of Wound Repair Materials

Moderate immunomodulation plays a crucial role in wound healing. During the initial stages of wound repair, the inflammatory response is essential for eliminating microorganisms and pathogens, thereby preventing wound infection. However, excessive inflammation can lead to prolonged non-healing. Dressings designed to promote wound healing through immunomodulation have a profound impact on various aspects of the healing process. They regulate processes such as mitochondria function, ROS levels, autophagy, ferroptosis, macrophage polarization, and cytokine production (refer to Table 1).

### 4.1. Mitochondria and ROS Regulation

**Figure 2:**
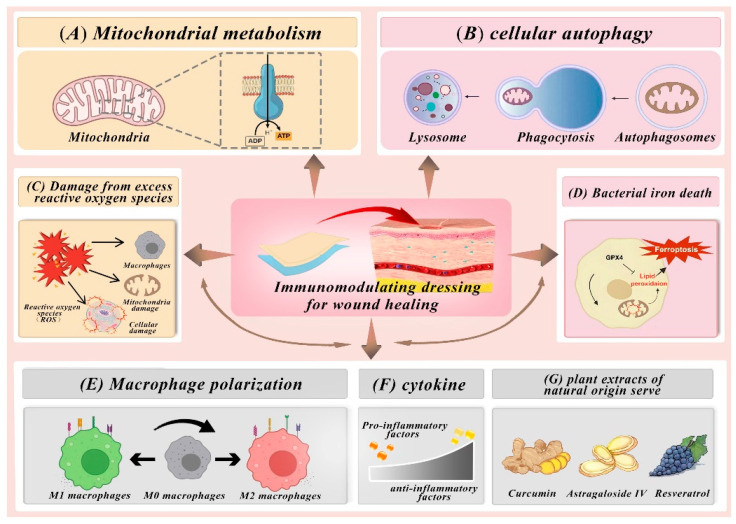
Immunomodulatory mechanisms of cutaneous wound dressings.

Cell proliferation, migration, angiogenesis, and immune responses involved in wound healing necessitate substantial energy consumption. Mitochondria serve as energy powerhouses by producing ATP through OxPhos and also play a critical role in maintaining appropriate pro-inflammatory and vascular responses during the early stages of wound healing by generating ROS [13,134,135]. However, the excessive accumulation of ROS can damage cell membranes and the ECM, thereby promoting the release of protein-degrading enzymes and inflammatory mediators, ultimately leading to delayed wound healing or scar formation during the inflammatory phase [136]. Additionally, there exists a close relationship between mitochondrial function and macrophage polarization. Mitochondrial dysfunction can induce oxidative stress by impairing bioenergetic efficiency, reducing angiogenesis, promoting ROS overproduction, affecting macrophage polarization, and ultimately resulting in abnormal inflammatory responses, thereby hindering wound healing and leading to chronic non-healing wounds (Figure 2A,C) [137,138,139,140].

**Figure 3:**
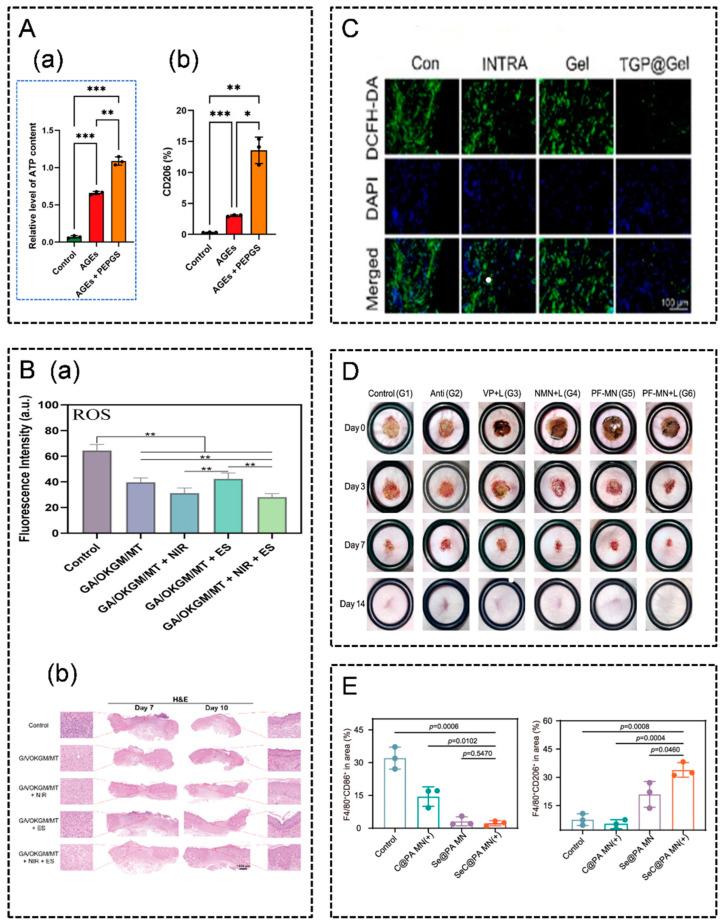
Studies of wound dressings that modulate mitochondria and ROS. (**A**) PEPGS promotes ATP and M2 macrophage production (* *p* < 0.05, ** *p* < 0.01 and *** *p* < 0.001). (**A**(**a**)) PEPGS was somewhat successful in restoring ATP production (AGEs + PEPGS vs. AGEs, 1.09 ± 0.06 μM vs. 0.66 ± 0.02 μM, *p* < 0.01). (**A**(**b**)) The PEPGS group (13.58 ± 2.13%) induced the conversion of RAW 264.7 cells into M2 macrophages [141]. (**B**) GA/OKGM/MT + NIR + ES reduces inflammatory cells by decreasing ROS (** *p* < 0.01). (**B**(**a**)) A reduction in the amount of ROS. (**B**(**b**)) Reduction in the amount of inflammatory cells (black scale bar: 1000 μm; white scale bar: 100 μm) [142] (**C**) TGP@CEC-HA hydrogel has excellent ROS scavenging ability (white scale bar: 100 μm) [147]. (**D**) PF-MN significantly promotes MRSA-infected diabetic wound healing (scale bar, 2 mm) [20]. (**E**) SeC@PA MN promotes M2 macrophage polarization [148].

Several immunomodulation-related wound dressings have been shown to promote wound healing by modulating mitochondria and ROS levels. Firstly, modulating the immune microenvironment of wounds through the restoration of mitochondrial function, the enhancement of mitochondrial metabolism, and scavenging excess ROS can enhance wound healing. Qi et al. developed a biodegradable poly(glycerol sebacate)-based multiblock hydrogel that releases glycerol, restoring mitochondrial function and enhancing metabolism, thus improving ATP synthesis and maintaining a mitochondrial redox state. This approach resulted in reduced ROS levels, accelerated macrophage polarization towards the M2 phenotype, the attenuation of the inflammatory response, and the promotion of wound healing in diabetic conditions (Figure 3A) [141]. Secondly, without directly acting on mitochondria, scavenging ROS alone can provide a potent anti-inflammatory effect and promote wound healing. Qiu et al. designed a hydrogel loaded with MXene@TiO nanosheets, which shortened the inflammatory phase of wounds by scavenging ROS and facilitated the transition to the proliferative phase (Figure 3B) [142]. Qu et al. developed a hydrogel patch that scavenges ROS, protecting recruited Tregs while inhibiting the differentiation of helper T17 cells, thus attenuating the inflammatory response and promoting wound healing [143]. Zhu et al. developed a metformin-laden CuPDA NPs composite hydrogel, which attenuated the inflammatory response by scavenging ROS and inhibiting NF-κB pathway activation, ultimately promoting wound healing [144]. Yang et al. developed a nanofiber mat loaded with various sugar alcohols and dopamine, and Zhang et al. developed a pH-responsive hydrogel, and all of these dressings provided a good anti-inflammatory effect by scavenging ROS, attenuating the inflammatory response and promoting wound healing [145,146]. Furthermore, scavenging excess ROS can inhibit scar formation and reduce the inflammatory response in wounds. Shu et al. developed a ROS-scavenging hydrogel by incorporating ROS-responsive moieties into carboxymethyl chitosan crosslinked with genipin. This hydrogel enhanced anti-inflammatory gene expression by effectively scavenging ROS from cells. It inhibited macrophage polarization towards the M1 type, attenuated the inflammatory response, mitigated cellular damage, reduced the expression of genes related to scar formation, and promoted scar-free wound healing [136]. Moreover, scavenging ROS can also promote wound healing by modulating macrophage polarization. Xiao et al. developed a nanofiber dressing loaded with Cu^2+^ and polydopamine, promoting the polarization of M1 macrophages to the M2 phenotype by scavenging excess ROS, alleviating inflammatory responses, and promoting wound healing [103]. Zhang et al. developed a hydrogel loaded with total glycosides of Paeonia lactiflora to promote macrophage polarization to the M2 phenotype by scavenging ROS (Figure 3C) [147]. Moreover, in addition to scavenging ROS to promote wound healing, moderate promotion of ROS generation can also play a role in certain cases. ROS exhibits antibacterial and anti-biofilm properties; moderate ROS generation can be beneficial for inhibiting infection and promoting wound healing. However, the barrier properties of biofilms can hinder the antimicrobial effectiveness of ROS. The physical penetration ability of microneedles provides a promising solution to this issue [148]. Zhang et al. designed a microneedle patch dressing called PF-MNs, which produce ROS to sterilize and destroy bacterial biofilm under laser irradiation, thereby promoting scarless wound healing (Figure 3D) [20].

It is evident that ROS exhibit a dual role in wound healing. A moderate increase in ROS during the early stages of wound formation can promote healing while preventing infection. However, sustained high levels of ROS can lead to a persistent inflammatory response, hindering wound healing. Balancing ROS levels by promoting ROS generation in the early stages of wound formation and removing excess ROS later on may be an effec-tive approach to promote wound healing. Zhou et al. developed a hydrogel based on a platinum nanozyme composite, which acted as a synergistic antimicrobial agent. Initially, it promoted the production of ROS, and subsequently removed excess ROS, thereby facili-tating the transition of the wound from the inflammatory phase to the proliferative phase and promoting wound healing [149]. Yang et al. developed a microneedle loaded with dopamine-coated hybrid nanoparticles containing selenium and chlorin e6. Initially, it promotes ROS production to destroy biofilms, and subsequently eliminates excess ROS, exerting an anti-inflammatory effect and promoting wound healing (Figure 3E) [148].

### 4.2. Impact of Autophagy on Immune Regulation in Wound Healing

Autophagy is a lysosome-dependent fundamental intracellular catabolic process that operates under high-stress conditions. It ensures that the internal and external environment of the cell remains uncompromised in the face of external damage or nutrient deficiencies, while simultaneously maintaining normal cellular physiological activity, cellular homeostasis, and cellular microenvironmental homeostasis (Figure 2B) [150,151,152]. Autophagy plays a crucial role in immune regulation during wound healing. Firstly, it promotes the accelerated repair of wounds by immune cells and functional cells, facilitating cell cycle regulation for self-renewal and ultimately promoting wound healing. Secondly, autophagy can activate inflammatory cells, exert anti-inflammatory, anti-infectious, and anti-oxidative effects, ensuring cell survival, promoting cell migration and proliferation, and regulating cellular activity and metabolism to support wound healing [152,153]. Additionally, macrophage autophagy contributes to wound healing by enhancing the immunological activity of wound tissue and suppressing the activity of inflammatory vesicles without inducing a stress response [154,155]. Moderate enhancement of autophagy can inhibit apoptosis and ROS production, mitigate oxidative stress injury, promote M2 macrophage polarization, and elevate levels of macrophages and proliferating cell nuclear antigens, thereby attenuating inflammatory responses and fostering wound healing [155,156,157]. However, excessive autophagy can impede angiogenesis and impair wound healing [158].

**Figure 4:**
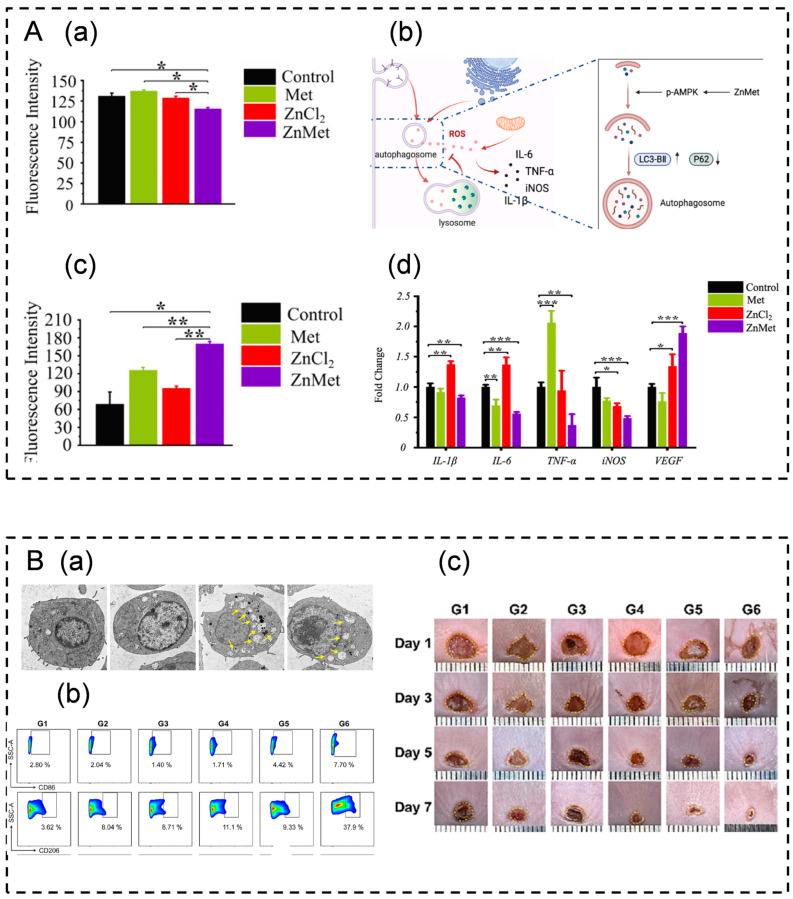
Studies on wound dressings that modulate autophagy. (**A**) ZnMet-PF127 attenuates inflammatory responses by promoting autophagy in NIH3T3 cells to inhibit ROS production (* *p* < 0.05, ** *p* < 0.01 and *** *p* < 0.001). (**A**(**a**)) ZnMet-PF127 scavenges ROS. (**A**(**b**)) Schematic diagram of the mechanism of ROS scavenging by ZnMet-PF127. (**A**(**c**)) ZnMet-PF127 promotes the formation of autophagosomes in NIH3T3 cells. (**A**(**d**)) ZnMet-PF127 reduces the level of pro-inflammatory factors [159]. (**B**) MGC NPs enhance the immunoreactivity of wound tissue by promoting macrophage autophagy. (**B**(**a**)) MGC NPs chitosan hydrogel polarizes M2 macrophages with a large number of autophagic vesicles. (**B**(**b**)) MGC NPs chitosan hydrogel promotes wound healing by activating the expression of M2 macrophages in the late wound healing phase. (**B**(**c**)) MGC NPs chitosan hydrogel promotes infected wound healing in mice [154].

Modulating cellular autophagy to enhance wound healing can be achieved through two primary strategies. Firstly, this can be achieved by activating autophagy and suppressing ROS production to mitigate inflammatory responses and stimulate angiogenesis, thus facilitating wound healing. Liu et al. developed a hydrogel dressing incorporating zinc-modified dimethylguanidine within a temperature-sensitive hydrogel Pluronic F127. This formulation promotes the growth and development of NIH3T3 cells by activating AMPK signaling and autophagy. Subsequently, it inhibits ROS production to mitigate the inflammatory response. Moreover, it induces the expression and secretion of vascular endothelial growth factor (VEGF), promoting angiogenesis and skin wound healing (Figure 4A) [159]. Secondly, wound healing can be bolstered by activating macrophage autophagy to enhance immunoreactivity. Kong et al. engineered a three-dimensional chitosan hydrogel loaded with melanin-glycine-C60 nanoparticles. This formulation augments the immunoreactivity of the wound tissue without inducing a stress response by stimulating macrophage autophagy, thereby promoting wound healing (Figure 4B) [154].

### 4.3. Effect of Iron Death on Immune Regulation in Wound Healing

**Figure 5:**
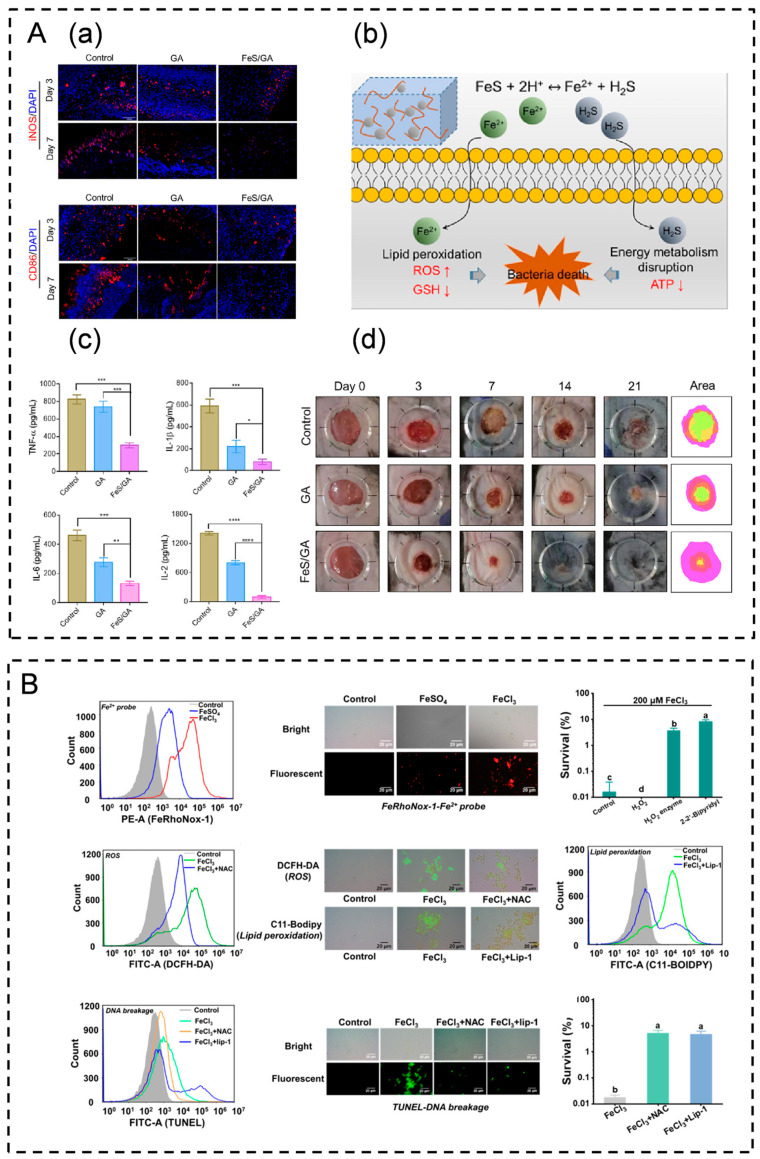
Studies on wound dressings that modulate bacterial iron death in wounds. (**A**) FeS/GA acts as an antibacterial agent by promoting iron death in *Staphylococcus aureus*. (**A**(**a**)) FeS/GA hydrogels decreased M1 macrophage activation and led to a shift to an M2 phenotype (white scale bar: 100 μm). (**A**(**b**)) Diagram of the mechanism by which FeS/GA hydrogels sterilize bacteria by inducing bacterial iron death. (**A**(**c**)) FeS/GA hydrogels reduce levels of pro-inflammatory factors (* *p* < 0.05, ** *p* < 0.01, *** *p* < 0.001, and **** *p* < 0.0001). (**A**(**d**)) FeS/GA hydrogels promote the healing of infected wounds in diabetic mice [162]. (**B**) Fe^3+^ in FeCl_3_-PB hydrogels successfully enters *P. aeruginosa* and triggers an elevated level of unstable Fe^2+^ in the cell, generating hydroxyl radicals that induce ROS production, lipid peroxidation, DNA damage, and iron death in *Pseudomonas aeruginosa* cells, which exerts an antimicrobial effect (statistical significance was denoted by different letters (*p* < 0.05) [163].

Iron death represents a distinct form of programmed cell death that differs from necrosis, apoptosis, pyroptosis, and autophagy. Its detrimental effects on skin wound healing stem from the generation of substantial amounts of ROS and lipid peroxidation products. These, in turn, induce lipid peroxidation damage, leading to structural and functional abnormalities and the cellular dysfunction of mitochondria [46,139,160,161]. However, this characteristic can be leveraged to induce ferroptosis in common wound infection pathogens such as *Staphylococcus aureus* and *Pseudomonas aeruginosa*, offering an effective antimicrobial approach without the risk of drug resistance (Figure 2D). Xu et al. developed a hydrogel loaded with FeS nanoparticles capable of disrupting the energy metabolism of *Staphylococcus aureus*. Through the sustained release of Fe^2+^ and H_2_S, this hydrogel induced a ferroptosis-like demise in bacteria. Furthermore, it downregulated pro-inflammatory cytokines, upregulated M2 phenotypic macrophages, facilitated fibroblast migration and proliferation, and promoted diabetic wound healing (Figure 5A) [162]. Similarly, Huang et al. developed a thermo-responsive hydrogel dressing by incorporating FeCl_3_ into a PVA-boric acid hydrogel. The inclusion of FeCl_3_ induced ferroptosis in *Pseudomonas aeruginosa* bacteria by triggering elevated levels of ROS, lipid peroxidation, and DNA damage. This process effectively served as a bactericidal agent, leading to bacterial iron death. Confirming its efficacy, a mouse wound infection model demonstrated that the hydrogel promoted the healing of infected wounds by treating underlying infections and facilitating pus removal (Figure 5B) [163].

### 4.4. Impact of Regulating Macrophage Behavior on Immune Modulation in Wound Healing

During the inflammatory phase of wound healing, wounds recruit circulating monocytes to the dermis, where they differentiate into pro-inflammatory M1-type macrophages under the influence of the inflammatory microenvironment. These M1-type macrophages dominate the early stages of wound healing, releasing nitric oxide (NO) to combat microorganisms and pathogens. Additionally, they express CD86 and secrete pro-inflammatory factors such as IFN-γ, IL-1, IL-6, and TNF-α, promoting pro-inflammatory responses and interacting with helper T cells 1 to regulate the inflammatory immune milieu [164,165]. As wound healing progresses from the inflammatory phase to the proliferative phase, macrophages transition from a pro-inflammatory M1 phenotype to an anti-inflammatory M2 phenotype. This transition mitigates the wound damage caused by inflammation and promotes tissue development, facilitating wound healing [166]. M2 macrophages play crucial roles by releasing anti-inflammatory cytokines such as IL-10, which induces neutrophil apoptosis, and by promoting the T helper 2 (Th2) response and activating the adaptive immune system, thereby regulating immune responses and attenuating inflammation. Furthermore, M2 macrophages secrete growth factors that foster tissue regeneration. A persistent presence of M1 macrophages in the wound, without polarization into M2-type macrophages, results in the sustained release of pro-inflammatory and cytotoxic molecules, perpetuating uncontrolled inflammation. Conversely, a deficiency in M2 macrophages leads to reduced levels of growth factors and an imbalance between pro-inflammatory and anti-inflammatory cytokines, ultimately resulting in impaired wound healing and the development of chronic non-healing wounds (Figure 2E) [164,165,167,168].

**Figure 6:**
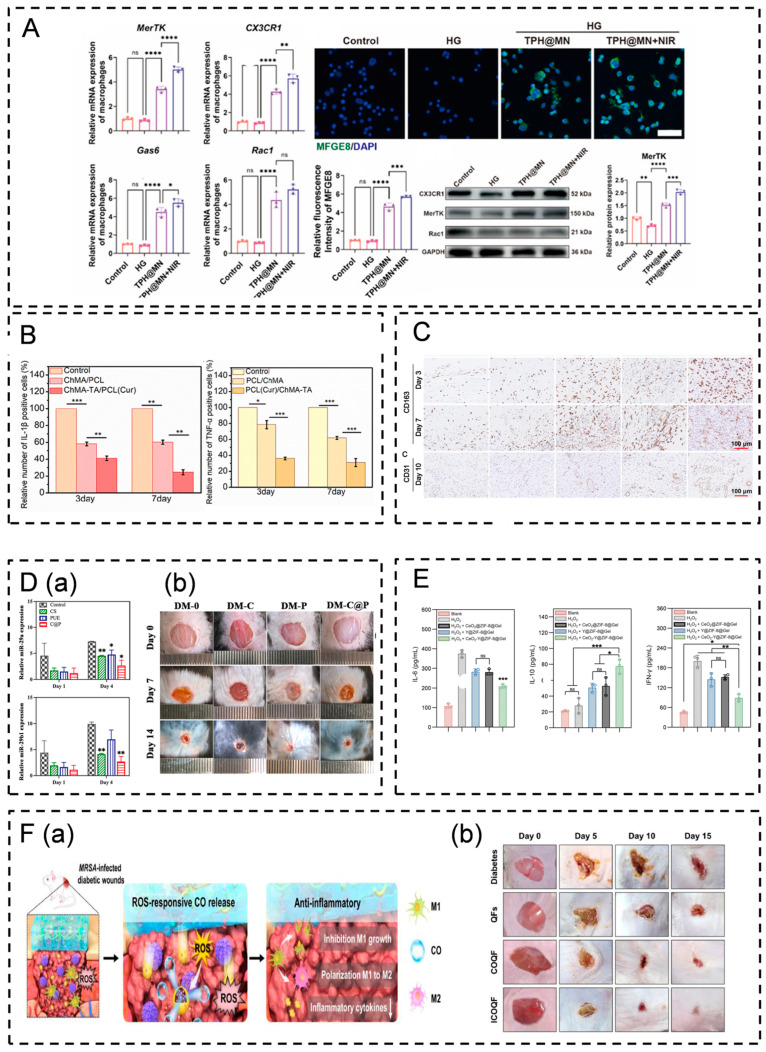
Studies on wound dressings that modulate macrophage behavior. (**A**) TPH@MN promotes the macrophage efferocytosis capacity, and the expression of MerTK, CX3CR1, Gas6, and Rac1, mRNAs associated with the role of efferocytosis, was significantly upregulated in macrophages after TPH@MN intervention (* *p* < 0.05, ** *p* < 0.01, *** *p* < 0.001, **** *p* < 0.0001; ns, not significant; white scale bar, 100 μm) [169]. (**B**) The ChMA/PCL nanofibrous membrane reduces pro-inflammatory factor levels (* *p* < 0.05, ** *p* < 0.01 and *** *p* < 0.005) [172]. (**C**) DNA-FKNa/Ag hydrogels promote M2 macrophage recruitment (red scale bar: 100 μm) [173]. (**D**) C@P attenuates the inflammatory response by reducing M1 macrophage polarization. (**D**(**a**)) C@P downregulates macrophage miR-29a/b1 expression (* *p* < 0.05, ** *p* < 0.01). (**D**(**b**)) C@P promotes wound healing in diabetic mice [79]. (**E**) Gel-QAS decreased the expression of pro-inflammatory factors secreted by M1 cells and increased the expression of anti-inflammatory factors secreted by M2 cells (* *p* < 0.05, ** *p* < 0.01, *** *p* < 0.001; ns, not significant) [76]. (**F**) ICOQF attenuates the inflammatory response by prompting macrophages to polarize from the M1 to the M2 type. (**F**(**a**)) ICOQF inhibits the proliferation of activated macrophages and promotes macrophage phenotypic polarization to fight inflammation. (**F**(**b**)) ICOQF promotes wound healing in diabetic mice infected with MRSA [181].

Regulating macrophage behavior can significantly enhance wound healing through various mechanisms. Firstly, boosting macrophage efferocytosis can mitigate the inflammatory response by clearing debris from senescent inflammatory cells, fostering macrophage polarization towards an anti-inflammatory phenotype, and promoting tissue repair and wound healing. Zhu et al. pioneered a heat-sensitive, anti-inflammatory photothermal microneedle hydrogel patch, which initiated tissue repair and facilitated wound healing by stimulating macrophage efferocytosis and eliminating senescent cells and debris (Figure 6A) [169]. Similarly, Liu et al. engineered a hybrid biomaterial to expedite the reprogramming of macrophages from a pro-inflammatory to an anti-inflammatory phenotype by enhancing macrophage efferocytosis, thereby accelerating diabetic wound healing [7].

Second, regulating macrophage recruitment at the wound site can profoundly influence the immunomodulation of wound healing. On the one hand, reducing macrophage infiltration and recruitment at the wound site can dampen the inflammatory response, thereby promoting wound healing. Li et al. pioneered a novel composite nanofiber (Figure 6B), Sun et al. developed a curcumin-functionalized electrospun fiber, and Pallavi Shyam Kaparekar et al. developed a nanocomposite scaffold, all of which facilitated wound healing by decreasing macrophage recruitment [170,171,172]. However, other studies have indicated that promoting macrophage recruitment in wounds can also accelerate wound healing. Liu et al. developed a sulfated galactofucan polysaccharide/poly(vinyl alcohol) hydrogel that fostered wound healing by recruiting macrophages to the wound site and upregulating their expression of CCL2, CCR2, and CCL22 mRNA, thereby expediting the transition from the inflammatory to the proliferative phase and promoting diabetic wound healing [117]. Furthermore, there is evidence suggesting that increasing M2 macrophage recruitment at the wound site can expedite wound healing. Gao et al. engineered an injectable DNA hydrogel carrying a fractalkine aptamer that facilitated M2 macrophage recruitment at the wound site by engaging G-coupled protein receptors and releasing endogenous chemokines, thereby accelerating the transition from the inflammatory phase to the proliferative phase of wound healing and promoting overall wound healing (Figure 6C) [173].

Third, regulating macrophage polarization can be highly effective in promoting wound healing, this is mainly reflected in the following points. ① Wound healing can be facilitated by inhibiting the activation or reducing the number of M1-type macrophages. Zeng et al. developed a chitosan hydrogel loaded with Puerariae, which promoted diabetic wound healing by inhibiting miR-29ab1 to decrease the polarization of M1 macrophages and attenuate inflammatory responses (Figure 6D) [79]. Li et al. developed an electrospun nanofiber patch loaded with Bunge-Radix Puerariae herbal compound, Ma et al. developed an itaconic acid-pluronic hydrogel, and Liu et al. developed a microneedle dressing loaded with macrophage liposomes and purpurolide C. All three formulations attenuated the inflammatory response by inhibiting the secretion of pro-inflammatory cytokines by M1 macrophages, thereby promoting tissue regeneration and wound healing [105,174,175]. ② The inflammatory response in wounds can be reduced, and tissue regeneration can be promoted by increasing the residence or polarization of M2-type macrophages to facilitate wound healing. Wang et al. developed a glycopeptide hydrogel capable of activating the TH2 immune response. Glycopeptide can be phagocytosed and processed by macrophages, leading to the differentiation of naïve T cells into TH2 cells, which promote wound healing by activating the TH2 immune response and increasing M2 macrophage residence, thereby enhancing angiogenesis and folliculogenesis in local wound tissues [176]. He et al. developed a hydrogel loaded with the Rho-associated protein kinase inhibitor, Y-27632, which inhibited the NLRP3 inflammatory pathway, downregulated the cGAS-STING pathway, and increased M2 macrophage polarization and anti-inflammatory factor levels, thereby attenuating the inflammatory response and promoting diabetic wound healing (Figure 6E) [76]. Gu et al. developed a fisetin silk fibroin hydrogel that can promote M2 macrophage polarization, attenuate inflammatory responses, promote tissue regeneration, and facilitate wound healing [177]. ③ Promoting M2 macrophage polarization while inhibiting M1 macrophage polarization could also attenuate the inflammatory response. Liu et al. developed a collagen scaffold for bone marrow mesenchymal stem cells, which could inhibit the secretion of the pro-inflammatory factors IL-1β, TNF-α, and MMP-9 and the polarization of M1 macrophages by inhibiting the inflammatory signaling pathway TNF-α/NF-κB, and at the same time could promote the secretion of the anti-inflammatory factors IL-1β, TNF-α and MMP-9 and the polarization of M2 the macrophage secretion of anti-inflammatory factors IL-10 and TGF-β3 secretion and polarization, which attenuate the inflammatory response and promote wound healing [111]. ④ Promoting the polarization of M1-type macrophages into M2-type macrophages is also an effective strategy to promote wound healing. Liu et al. developed a sponge dressing loaded with exosome-mimicking nanovesicles, Qi et al. developed an AuPt@melanin-incorporated hydrogel, Zhang et al. developed a gallium/glycyrrhizic hydrogel, and Wang et al. developed a PAA-based cohesive hydrogel, all of which attenuated the inflammatory response and promoted wound healing by inducing the polarization of macrophages from M1-type to M2-type [128,178,179,180]. ⑤ Inhibiting the proliferation of activated macrophages while promoting the polarization of M1 macrophages to the M2 type also promotes wound healing. Chen et al. developed a CO gas therapy-based hydrogel that attenuated the inflammatory response and promoted wound healing by inhibiting the proliferation of activated macrophages while promoting the polarization of macrophages from the M1 to the M2 type (Figure 6F) [181].

In conclusion, enhancing the population of M2 macrophages by inhibiting M1 macrophage activity and numbers, and promoting their polarization towards the M2 phenotype, appears to be a promising strategy for promoting wound healing. However, the specific mechanisms through which M2 macrophages facilitate wound repair require further investigation. Studies have delineated four distinct subtypes of M2 macrophages, each playing unique roles: M2a promotes fiber regeneration, M2b exhibits anti-inflammatory properties, M2c facilitates ECM cleavage, and M2d supports vascular regeneration. It is important to note that sustained activation of M2 macrophages or abnormal phagocytosis by M2c may potentially lead to scar formation [165,182]. Hence, in the future, it is crucial to explore methods for accurately regulating different subtypes of M2 macrophages to optimize wound healing outcomes.

### 4.5. Cytokine Regulation

Cytokines that affect wound healing by participating in immune regulation are mainly inflammatory chemokines, pro-inflammatory cytokines, and anti-inflammatory cytokines. Inflammatory chemokines, such as MCP-1 and IL-8, are secreted by immune cells and specific cells residing at the injury site [183]. Pro-inflammatory cytokines, including TNF-α, IL-1β, and IL-6, are released by M1 macrophages [184]. M2 macrophages produce numerous anti-inflammatory cytokines (e.g., IL-4, IL-10), epidermal growth factor, VEGF, and fibroblast growth factor, with anti-inflammatory cytokines primarily serving to dampen inflammation [185]. The inflammatory response in wound healing normally subsides after a few days through the release of inhibitory cytokines from M1-polarized to M2 macrophages [186]. However, excessive inflammatory chemokines and pro-inflammatory cytokines can prolong inflammation in the wound. Excessive pro-inflammatory cytokines hinder macrophage polarization to the M2 type, while elevated levels of M1 macrophages secrete more pro-inflammatory cytokines, creating a reinforcing cycle that contributes to the formation of chronic non-healing wounds (Figure 2F) [165,187].

**Figure 7:**
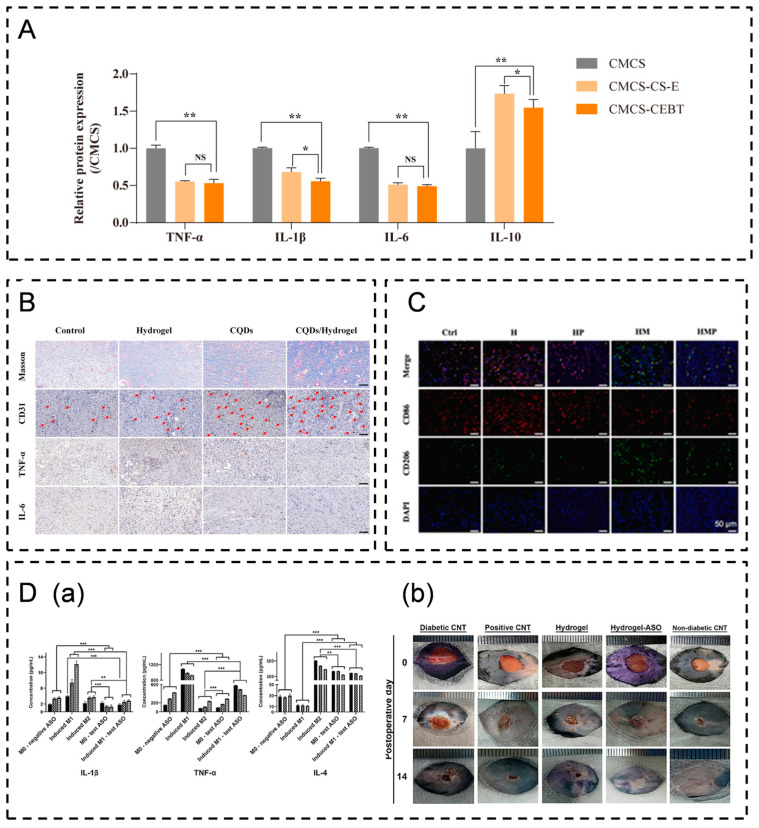

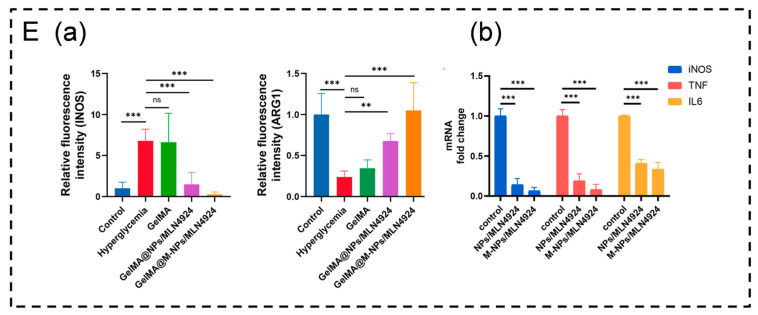
Studies on wound dressings that modulate cytokines. (**A**) CMCS-CEBT reduced levels of pro-inflammatory factors TNF-α, IL-1β, IL-6, and IL-10 (* *p* < 0.05, ** *p* < 0.01; NS, not significant) [189]. (**B**) CQDs/hydrogels reduce levels of pro-inflammatory factors TNF-α and IL-6 and promote collagen deposition and angiogenesis (scale bar: 100 μm) [192]. (**C**) HMP hydrogel promotes macrophage polarization from M1 to M2 (scale bar: 50 μm) [194]. (**D**) Man-PEI-ASO hydrogel promoted M2 macrophage polarization by decreasing the levels of inflammatory factors and increasing the levels of anti-inflammatory factors. (**D**(**a**)) Man-PEI-ASO hydrogel reduced the expression of pro-inflammatory factors IL-1β and TNF-α and increased the expression of anti-inflammatory factor IL-4 (** *p* < 0.01 and *** *p* < 0.001; ns, not significant). (**D**(**b**)) Man-PEI-ASO hydrogel promotes wound healing in diabetic mice [186]. (**E**) M-NPs/MLN4924 hydrogels promoted M2 macrophage polarization by decreasing levels of inflammatory factors and increasing levels of anti-inflammatory factors (** *p* < 0.01, *** *p* < 0.001). (**E**(**a**)) M-NPs/MLN4924 hydrogels inhibited macrophage M1 polarization and promoted their shift to an M2 repair phenotype. (**E**(**b**)) M-NPs/MLN4924 reduced TNF-α and IL-6 secretion [193].

Modulating cytokine expression, production, and release patterns can significantly enhance wound healing through three key mechanisms. Firstly, balancing pro- and anti-inflammatory factors is critical for mitigating inflammation and promoting recovery. For example, Wang et al. developed a cytokine hydrogel incorporating the anti-inflammatory cytokine IL-33 within a DNA hydrogel matrix. This formulation localized regulatory T cells and group 2 innate lymphoid cells to the wound site, facilitating the transition of M1-type macrophages to the M2 phenotype and enhancing ROS clearance. These actions collectively reduced the inflammatory response and improved diabetic wound healing [188]. Similarly, Shang et al. developed a bioactive composite hydrogel that augmented the expression of anti-inflammatory factors, thereby promoting wound healing (Figure 7A) [189]. Additionally, Irfan Khan et al. engineered a collagen scaffold loaded with mesenchymal stem cells, while Zhao et al. designed a nanocascade reactor, both of which promoted wound healing by upregulating the expression of anti-inflammatory factors TGFβ1 and IL-10, respectively [112,190]. Subsequently, an anti-inflammatory effect and promotion of wound healing can be achieved by inhibiting the expression of pro-inflammatory factors. Xian et al. formulated a hybrid hydrogel wound dressing, exhibiting potent anti-inflammatory effects and promoting wound healing by downregulating the expression of pro-inflammatory cytokines IL-1β and TNF-α [191]. Likewise, Wang et al. introduced a novel bioinspired radial-loaded porous zinc-based metal–organic framework composite sponge, which dampened the inflammatory response by downregulating inflammatory cytokines while facilitating wound healing through antimicrobial and antioxidant properties [121]. In a similar vein, Lu et al. engineered a hydrogel loaded with carbon quantum dots (Figure 7B), Pejman Ghelich et al. developed a microneedle dressing loaded with recombinant human proteoglycan 4, and Zeng et al. created a macrophage-mimetic membrane encapsulated with nanoparticles (Figure 7E). These innovations effectively promoted wound healing by reducing the levels of pro-inflammatory factors TNF-α and IL-6, thereby mitigating the inflammatory response [118,192,193]. Furthermore, lowering levels of pro-inflammatory cytokines while elevating levels of anti-inflammatory cells can also expedite wound healing. Tu et al. innovated a nano-enzyme-containing hydrogel that effectively reduced levels of the inflammatory chemokine CXCL-1 and pro-inflammatory cytokines IL-1β and TNF-α. Simultaneously, it elevated levels of anti-inflammatory cytokines such as IL-10 and IL-4. This dual action promoted the polarization of M2 macrophages and dampened the inflammatory response. Additionally, the hydrogel exhibited ROS scavenging and antibacterial abilities, facilitated collagen deposition, angiogenesis, and ultimately accelerated wound healing (Figure 7C) [194]. Moreover, Zhang et al. developed an injectable bioactive nanoglass hydrogel with a self-healing ability, which suppressed the level of pro-inflammatory factor TNF-α while increasing levels of anti-inflammatory factors IL-4 and IL-10, thereby attenuating the inflammatory response and promoting wound healing [195]. Similarly, Yang et al. engineered a magnesium/gallic acid bio-MOFs-laden carbonized mushroom aerogel, and Li et al. devised a hydrogel comprising peptide C8G2 and β-glucan-peptide, both serving as dressings, effectively modulating inflammatory cytokine levels to attenuate the inflammatory response and promote wound healing [77,88]. Secondly, wound healing can also be facilitated by modulating cytokine-related genes. Moein Amoupour et al. devised a macrophage “Suppressor of Cytokine Signaling 3” gene-expression-inhibiting hydrogel, which promotes wound healing by reducing levels of pro-inflammatory cytokines IL-1β and TNF-α. Through diminishing the secretion of these cytokines and augmenting the secretion of anti-inflammatory cytokine IL-4, the hydrogel encourages M2-type macrophage polarization, thereby mitigating inflammatory responses and fostering diabetic wound healing (Figure 7D) [186]. Thirdly, altering the pattern of cytokine production and release represents a promising strategy for enhancing wound healing. Mao et al. developed a “cytokine reservoir” hydrogel that initially recruits macrophages and actively stimulates mannose receptors. This stimulation prompts macrophages to secrete substantial amounts of pro-regenerative cytokines while polarizing toward the M2 type. Subsequently, the hydrogel collects and stores these pro-regenerative cytokines, thereby modifying the cytokine release pattern to sustain their beneficial effects throughout the wound healing process [196]. Upon hydrogel degradation, these pro-regenerative cytokines are released, and the quality and duration of release can be regulated, substantially contributing to inflammation reduction, tissue regeneration, and offering considerable promise as a wound healing dressing.

**Table 1:** Key immunomodulatory mechanisms and outcomes of wound healing biomaterials.

Types	Composition	Cell/Animal	Model	Machine	Results	Ref.
Hydrogels	PEPGS hydrogel	RAW264.7 cells/ HUVECs/rats	Diabetic wound model (rats)	Restoration of mitochondrial function	ROS ↓, M2 macrophage polarization ↑, ATP ↑	[141]
	GA/OKGM/MT hydrogel	Rats	Dorsal skin wound model (rats)	Eliminated excessive ROS	Bacteria ↓, ROS ↓, Inflammation ↓, proliferation ↑	[142]
	RSG-CCL22 hydrogel Patch	Mice lymphocytes/ splenocytes/mice	Diabetic full-thickness skin defect model (mice)	Eliminated excessive ROS	ROS ↓, Th17 differentiation ↓, local inflammation ↓.	[143]
	Met@CuPDA NPs/ HG hydrogel	Rats	A diabetic full-thickness skin defect infection model (rats)	Eliminated excessive ROS	ROS ↓, iNOS ↓, arginase ↑, inflammatory response ↓.	[144]
	ZC-QPP hydrogels	Rats	*Staphylococcus aureus* skin infection wound model (rats)	Eliminated excessive ROS	Bacterial membranes ↓, ROS ↓, inflammation ↓.	[146]
	CT-X gels	RAW264.7 cells/L929 cells/HUVECs/rats	Square wound model of diabetic full-thickness excised skin (rats)	Eliminated excessive ROS	Bacterial membranes ↓, ROS ↓, inflammation ↓.	[136]
	CEC-HA hydrogel	Fibroblasts/ HUVECs/ L929 cells/rats	Diabetic full-thickness dorsal skin defect model (rats)	Eliminated excessive ROS	ROS ↓, M2 macrophage polarization ↑, M1 macrophage polarisation to M2 phenotype ↑, pro-inflammatory cytokines ↓	[147]
	PFOB@PLGA@Pt/ GelMA/ODex hydrogel	HUVECs/L929 cells/RAW264.7 cells/ mices	Diabetic wound model with bacterial infection (mices)	ROS is first generated and subsequently cleared	Bacteria ↓, M1 macrophages ↓, inflammation ↓, proliferation ↑	[149]
	ZnMet-PF127 hydrogel	Human embryonic fibroblasts/HUVECs/ mice	Traumatic skin defect and skin burn model (mice)	Promote cellular autophagy	ROS ↓, inflammation ↓	[159]
	MGC NPs chitosan hydrogel	NCTC clone 929 cells/ dendritic cells/RAW 264.7 cells/mice	Infected wounds on the back (mice)	Promotion of macrophage autophagy	IL-1β ↑, TLR-1 ↑, M2 macrophage autophagy ↑, migration of epithelial cells ↑	[154]
	FeS/GA hydrogels	RAW264.7 cells/3T3 fibroblasts cells/mice	Diabetic wound infection model (mice)	Induction of iron death in bacteria	Bacteria ↓, M2 macrophages ↑, pro-inflammatory cytokines ↓	[162]
	FeCl_3_-PB hydrogels	Mice	Clinic Isolate infected full-thickness skin wound model (mice)	Induction of iron death in bacteria	Bacteria ↓, IL-6 ↓, TNF-α ↓, IL-1β ↓	[163]
	Gel@fMLP/SiO_2_-FasL	Neutrophils and macrophages of mice bone marrow origin/mice	Diabetic skin wound healing model (mice)	Boosting macrophage efferocytosis	Macrophage efferocytosis ↑, apoptotic neutrophils ↓, M2 macrophage polarization ↑	[7]
	DPH_20_ hydrogel	RAW264.7 cells/L929 cells/HUVECs/mice	Dorsal full-thickness excisional skin wound model (mice)	Regulation of macrophage recruitment	macrophage recruitment ↑, inflammation ↓, proliferation ↑	[117]
	DNA-FKNa/Ag hydrogel	NIH3T3 cells/rats	MRSA infection model for total skin defects (rats)	Regulation of macrophage recruitment	M2 macrophage recruitment ↑, inflammation ↓, proliferation ↑	[173]
	Chitosan@Puerarin hydrogel	RAW264.7 cells/mice	Type 1 diabetic whole skin wound model (mice)	Regulation of macrophage typing.	Macrophage ↓, M1 macrophage polarization ↓, IL-1β ↓, TNF-α ↓	[79]
	FIA hydrogel	RAW264.7 cells/L929 cells/HUVECs/mice	MRSA-injured full-thickness skin defect wound model (mice)	Regulation of macrophage typing	Bacteria ↓, M1-type macrophages ↓, pro-inflammatory cytokine levels ↓, inflammation ↓	[174]
	GRK hydrogel	Mice bone marrow macrophage/mice	Mice spleen lymphocytes/Dorsal full-thickness wound model (mice)	Regulation of macrophage typing	TH2 cell differentiation ↑, M2 macrophage residency ↑, type 2 immune response ↑, proliferation ↑	[176]
	CeO2–Y@ZIF-8@Gel	L929 cells/Thp-1 monocyte/Human epidermal keratinocyte cells/ hUMSC/HUVEC/ mice	Diabetic full-thickness skin wounds (mice)	Regulation of macrophage typing	Macrophage infiltration ↓, unfractionated macrophages polarized to M2 phenotype ↑, anti-inflammatory cytokines ↑	[76]
	Fis@ZnO@SFMA	RAW264.7 cells/L929 cells/mice	Whole skin wound model of diabetic infection	Regulation of macrophage typing	Bacteria ↓, M2 macrophage polarization ↑, IL-6 ↓, TNF-α ↓	[177]
	GHM3 hydrogel	RAW264.7 cells/HUVECs/rats	Diabetic skin/foot ulcers wound model (rats)	Regulation of macrophage typing	ROS ↓, M2 macrophage polarization ↑, M1 macrophages polarized to M2 phenotype ↑	[178]
	Ga/GA hydrogels	RS1 cells/RAW264.7 cells/HUVECs/mice	Infected pressure ulcer injury model (mice)	Regulation of macrophage typing	Biofilm ↓, M2 macrophage polarization ↑, M1 macrophages polarized to M2 phenotype ↑	[179]
	PAA-IPEG/TA coacervate hydrogel	RAW264.7 cells/rats	A model of full-layer skin infection on the back (rats)	Regulation of macrophage typing	M1 macrophages polarized to M2 phenotype ↑, bacteria ↓, Bacteria ↓, haemostasis time ↓, healing ↑	[180]
	ICOQF hydrogel	RAW264.7 cells/HUVECs/mice	MRSA-infected diabetic whole skin wound model (mice)	Regulation of macrophage typing	ROS ↓, CO ↑, M1 macrophage polarization to M2 phenotype ↑, macrophage proliferation ↓	[181]
	IL-33-cytogel hydrogel	Human epidermal keratinocyte cells/mice	Diabetic full-thickness skin wound model (mice)	Regulation of pro-/anti-inflammatory factor balance	ROS ↓, ilc2 ↑, M2 macrophages ↑, treg ↑, M1 macrophage polarization to M2 phenotype ↑	[188]
	CMCS-CEBT hydrogel	HUVECs/L929 cells/RAW264.7 cells/mice	Diabetes mellitus whole skin damage model/Skin burn wound model (mice)	Regulation of pro-/anti-inflammatory factor balance	TNF-α ↓, IL-1β ↓, IL-6 ↓, IL-10 ↑, inflammation ↓, wound healing ↑	[189]
	FE/HA-MA hydrogel	HUVECs/RAW264.7 cells/mice	Diabetic full-thickness skin wound model (mice)	Regulation of pro-/anti-inflammatory factor balance	TNF-α ↓, IL-1β ↓, VEGF-A ↑, inflammation ↓, healing ↑	[191]
	CQDs/hydrogel	HUVECs/RAW264.7 cells/rats	A model of MRSA-infected whole skin defects (rats)	Regulation of pro-/anti-inflammatory factor balance	NO ↓, TNF-α ↓, IL-6 ↓, inflammation ↓, angiogenesis ↑	[192]
	GelMA@M-NPs/MLN4924 hydrogel	HUVECs/L929 cells/RAW264.7 cells/mice	Diabetic total excision of skin wounds model (mice)	Regulation of pro-/anti-inflammatory factor balance	ROS ↓, NF-κB ↓, M1 macrophages ↓, TNF-α ↓, IL-6 ↓	[193]
	HMP hydrogel	L929 cells/rats	MRSA-infected diabetic total excisional skin wound (rats)	Regulation of pro-/anti-inflammatory factor balance	MRSA ↓, M2 macrophages ↑, TNF-α ↓, IL-1β ↓, IL-6 ↓, CXCL-1 ↓, IL-4 ↑, IL-10 ↑	[194]
	FABA hydrogel	L929 cells/RAW264.7 cells/mice	MRSA infected skin wound model (mice)	Regulation of pro-/anti-inflammatory factor balance	Bacteria ↓, TNF-α ↓, IL-4 ↓, IL-10 ↓, inflammation ↓, healing ↑	[86]
	BGA/C_8_G_2_ hydrogel	Mice embryonic fibroblasts/HUVECs/RAW264.7 cells/rats	MRSA infected skin wound model (rats)	Regulation of pro-/anti-inflammatory factor balance	Bacteria ↓, M2 macrophage polarization ↑, NO ↓, IL-6 ↓, TNF-α ↓, IL-10 ↑	[77]
	Man-PEI-ASO hydrogel	RAW264.7 cells/mice	Diabetic total excision of skin wounds model (mice)	Regulation of cytokine-related genes	IL-4 ↑, IL-10 ↑, TNF-α ↓, IL-1β ↓, M2 macrophage polarization ↑, M1 macrophage ↓	[186]
	EPL-DOPA hydrogel	HUVECs/RAW264.7 cells/mice	Diabetic total excision of skin wounds model (mice)	Altered patterns of cytokine production and release	iNOS ↓, TNF-α ↓, IL-10 ↑, M2 macrophage polarization ↑	[196]
Nanofiber dressings	Xyl@PVA/DA-Ag Nanofiber Mats	No	No	Eliminated excessive ROS	ROS ↓, Bacteria ↓, inflammation ↓, pain and local heat ↓, burn wounds healing ↑	[145]
	Cu^2+^-PDA-PCL nanofibrous dressing	RAW 264.7 cells/HUVECs/rats	MRSA-infected full-thickness skin wound model (rats)	Eliminated excessive ROS	MRSA ↓, macrophage M1 polarization ↑. M1 macrophages polarized to M2 macrophages ↑	[103]
	ChMA/PCL nanofibrous membrane	L929 cells/mice	Full-layer skin defect wound model (mice)	Regulation of macrophage recruitment	Macrophage infiltration ↓, pro-inflammatory cytokine levels ↓, inflammation ↓	[172]
	Gel/PCL-Cur3 fibrous membrane	Natural human dermal fibroblasts/RAW 246.7 cells/Rats	Subcutaneous injection model (rats)	Regulation of macrophage recruitment	Bacteria ↓/macrophage recruitment ↓, inflammation ↓, TNF-α ↓, IL-6 ↓	[170]
	GA-CSNPs nanocomposite scaffold	Rats	Full dorsal open diabetic incision model (rats)	Regulation of macrophage recruitment	Macrophage recruitment ↓, inflammation ↓, regeneration ↑	[171]
	Gel/PLLA-SRHC nanofibrous textiles	Human dermal fibroblasts/RAW264.7 cells/mice	The classic type I diabetes mellitus whole-layer wound model (mice)	Regulation of macrophage typing	M1 Macrophage activation ↓, pro-inflammatory factors ↓	[105]
Microneedles	PF-MNs	Male BALB/c mice	Acute wound/diabetic infected wound model (mice)	Promote ROS generation appropriately	Biofilm ↓, pro-inflammatory cytokines ↓, inflammation ↓, proliferation ↑, scarring ↓	[20]
	SeC@PA and SeC@PA MN bandage	HUVECs/RAW264.7 cells/mice	Diabetic infected/non-infected whole skin wound model (mice)	ROS is first generated and subsequently cleared	Biofilm ↓, M2 macrophage polarization ↑, inflammation ↓, healing ↑	[148]
	TPH@MN patches	L929 cells/RAW 246.7 cells/C2C12 cells/HUVECs/mice	Chronic diabetic wound model (mice)	Boosting macrophage efferocytosis	Macrophage efferocytosis ↑, inflammatory cell debris ↓, tissue repair ↑	[169]
	Purpurolide C-based microneedle	Mice peritoneal macrophages/RAW 246.7 cells/mice	Type 2 Diabetes Wound Model (mice)	Regulation of macrophage typing	M1 macrophage activation and polarization ↓, pro-inflammatory cytokines ↓, MD2 and MYD88 protein ↓	[175]
	FE/HA–MA Hybrid Hydrogels	HUVECs/RAW264.7 cells/mice	Diabetic Wound Model (mice)	Regulation of pro-/anti-inflammatory factor balance	TNF-α ↓, IL-1β ↓, VEGF-A ↑, inflammation ↓, healing ↑	[118]
Collagen scaffolds	CBS-MSCs Collagen scaffolds	RAW264.7 cells/bone marrow stem cells of mice/mice	Whole skin defect model (mice)	Regulation of macrophage typing	MMP-9 ↑, IL-1β ↓, TNF-α ↓, IL-10 ↑, TGF-β3 ↑, M1 macrophages polarized to M2 phenotype ↑	[111]
	Collagen scaffolds loaded with MSC	Rats	Hypoxic wound model (rats)	Regulation of pro-/anti-inflammatory factor balance	TGFβ1 ↑, inflammation ↓, wound healing ↑	[112]
Sponge dressings	CTM collagen sponge	HUVECs/huc-mscs/ L929 cells/RAW 246.7 cells/rats	Diabetic skin trauma model (rats)	Regulation of macrophage typing	Macrophages polarized to M1 type ↓, polarized to M2 type ↑, M1 macrophages polarized to M2 ↑	[128]
	SPCP/Zn sponge	L929 cells/rats	Whole skin defect wound model (rats)	Regulation of pro-/anti-inflammatory factor balance	Bacteria ↓, hemostasis ↑, TNF- ↓, IL-6 ↓, VEGF ↑	[121]
Aerogels	QMOFs-PCMA	L929 cells/RAW 246.7 cells/rats	A model of *S. aureus*-infected diabetic total skin defects (rats)	Regulation of pro-/anti-inflammatory factor balance	ROS ↓, M1 macrophages polarized to M2 ↑, TNF-α ↓, IL-1β ↓, IL-10 ↑, TGF-β ↑	[91]
Nanomaterials	MACNL	NIH/3T3 cells/HUVECs/mice	MRSA-infected diabetic total excisional skin wound (mice)	Regulation of pro-/anti-inflammatory factor balance	NO ↑, M2 macrophage polarization ↑, IL-10 ↑, TGF-β1 ↑, VEGF ↑	[190]

↑: increased; ↓: decreased.

## 5. Biomaterials Loaded with Plant Extracts to Modulate the Immune Microenvironment

Previously, the conventional approach involved incorporating antibiotics into dressings to prevent and treat infections and excessive inflammation in wounds. However, antibiotics are prone to developing resistance and may entail long-term adverse effects [197,198]. In contrast, plant extracts of natural origins serve as ideal alternatives to antibiotics. They do not foster resistance, exhibit high biocompatibility and safety, and can foster wound healing by modulating the immune response. Several plant extracts suitable for loading into dressings are outlined below, including Curcumin, Astragaloside IV, and Resveratrol (Figure 2G). Additionally, dressings infused with extracts from aloe vera, birch, and moringa leaf have demonstrated efficacy in promoting wound healing through immunomodulation [199,200,201,202].

### 5.1. Curcumin

Curcumin, a natural polyphenol primarily extracted from the powdered rhizome of turmeric, possesses notable anti-inflammatory, antibacterial, and antioxidant properties, effectively promoting wound healing [203]. Curcumin exerts its anti-inflammatory function through various mechanisms: First, it induces the apoptosis of inflammatory cells and reduces the expression of pro-inflammatory cytokines IL-1 and TNF-α. Second, it recruits and stimulates M2 macrophages to secrete anti-inflammatory cytokines. Third, it inhibits the activation of the NF-κB pathway by suppressing the activities of AKT, IKK, and PI3K. Fourth, it attenuates angiotensin II-induced inflammatory responses by enhancing peroxisome proliferator-activated receptor-γ activity. Furthermore, curcumin inhibits the production of the TLR4-MD2 signaling complex by binding to MD2, thereby mitigating inflammation. Simultaneously, curcumin scavenges excess ROS, inhibits oxidation-related transcription factors, and enhances the production and activity of antioxidant enzymes and their components. Additionally, curcumin exhibits potent antimicrobial properties and enhances cell proliferation and migration, thereby facilitating wound healing through collagen deposition, granulation, and wound re-epithelialization [204].

In recent years, a plethora of curcumin-loaded wound dressings have emerged and been clinically utilized to promote wound healing through immunomodulation. First, curcumin facilitates macrophage polarization and cytokine modulation to mitigate inflammation. For instance, Fan et al. engineered a curcumin-loaded hydrogel to mitigate inflammatory responses via ROS scavenging, the downregulation of the pro-inflammatory cytokine IL-1β, the promotion of M1-type macrophage polarization to the M2-type, the upregulation of CD31 expression, and the enhancement of collagen deposition and angiogenesis, thereby fostering wound healing [205]. Second, curcumin also mitigates inflammatory responses induced by bacterial infections. Zhang et al. devised a hyaluronan protein membrane laden with curcumin, the antimicrobial peptide KR-12, and AgNPs. This dressing inhibited inflammatory responses by modulating macrophage polarization, eradicating methicillin-resistant *Staphylococcus aureus* (MRSA), and inhibiting inflammation to promote wound re-epithelialization and angiogenesis, expediting wound healing in infected wounds [206]. Third, curcumin reduces the influx of immune cells and downregulates the inflammatory response in wounds. Zhang et al. developed a curcumin-loaded glycosaminoglycan-based hydrogel, which facilitated wound healing in diabetic mice by reducing the influx of immune cells at the wound site, downregulating the inflammatory response, and regulating the wound microenvironment from multiple perspectives [207]. Fourth, curcumin attenuates the inflammatory response by binding to key residues of inflammatory proteins. Hitesh Chopra et al. formulated a curcumin-loaded chitosan hydrogel that attenuated the inflammatory response and promoted wound healing by binding to key residues of inflammatory proteins active in wound healing [208].

### 5.2. Astragaloside IV

Astragaloside IV (AS-IV) constitutes one of the principal active components of Astragalus [208]. AS-IV exhibits anti-inflammatory, antioxidant, and immunomodulatory properties. It promotes the polarization of macrophages towards the M2 phenotype, upregulates the anti-inflammatory cytokine TGF-β, and downregulates pro-inflammatory cytokines at the inflammatory site, thus mitigating the inflammatory response of wounds [25]. The anti-inflammatory effects of AS-IV are multifaceted. Firstly, AS-IV’s involvement in the PI3K/AKT/mTOR signaling pathway, as demonstrated by Xiong et al., enhances its ability to mitigate the expression of inflammatory factors. Secondly, AS-IV exhibits antioxidant properties, as indicated by Gao et al., who found that it suppresses the overexpression of ROS and pro-inflammatory cytokines IL-6 and IL-8. This action is mediated through activation of the TGF-β/Smad signaling pathway, which also boosts levels of anti-inflammatory cytokines IL-10 and TGF-β1. These mechanisms collectively contribute to reducing inflammation and promoting healing in diabetic wounds [209,210]. Furthermore, AS-IV facilitates tissue repair and wound healing by stimulating the production of various cytokines, growth factors, collagen, MMPs, and the ECM, thereby promoting the proliferation and migration of keratinocytes and other skin cells [25,204,210].

In recent years, several AS-IV-loaded wound dressings have been developed and applied clinically, leveraging its immunomodulatory properties to promote wound healing. These dressings primarily mitigate inflammatory responses through their antimicrobial, anti-inflammatory, and antioxidant attributes. Zhao et al. innovated an injectable hydrogel incorporating AS-IV, which demonstrated effective antimicrobial, anti-inflammatory, and antioxidant characteristics. This formulation facilitated the scarless repair of MRSA-infected wounds by modulating inflammation, balancing the ratio of type I to type III collagen, and promoting angiogenesis and the formation of granulation tissue [211]. Similarly, Zhang et al. devised a silk fibroin/gelatin electrospun nanofiber wound dressing infused with AS-IV, which diminished neutrophils at the wound site, attenuated the inflammatory response, promoted angiogenesis and granulation tissue formation, thereby expediting wound healing [212]. Secondly, AS-IV-loaded dressings also alleviate inflammation by promoting macrophage polarization and reducing pro-inflammatory factors. Liu et al. developed an AS-IV-loaded nano-spray wound dressing with potent antimicrobial properties, effectively inactivating *Staphylococcus aureus* and *Escherichia coli*. This formulation promoted macrophage polarization towards an M2 phenotype, suppressed the expression of the pro-inflammatory factor TNF-α, facilitated ECM deposition, promoted vascular remodeling, and ultimately facilitated wound healing [25]. Similarly, Zha et al. designed an antimicrobial bilayer hydrogel loaded with AS-IV, promoting macrophage polarization to an M2 phenotype and scavenging of ROS during the inflammatory and early proliferative phases through AS-IV release, fostering angiogenesis, and facilitating rapid and scarless wound healing [213].

### 5.3. Resveratrol

Resveratrol, a non-flavonoid polyphenolic compound abundant in the skins and seeds of red grapes, possesses a myriad of properties such as anti-inflammatory, antimicrobial, antioxidant, and angiogenic capabilities that facilitate wound healing. On the one hand, resveratrol impedes macrophage infiltration and promotes their polarization to the M2 phenotype via the PI3K/AKT pathway, elevating the levels of the anti-inflammatory factor IL-10 while reducing pro-inflammatory factors such as COX-2 and its derivatives, PGE2, TNF-α, IL-1β, and iNOS. This action effectively mitigates the inflammatory response in wounds, likely through the mediation of the NF-κB and MAPK signaling pathways. Furthermore, resveratrol exhibits antioxidant properties by inhibiting ROS production through the upregulation of antioxidants. On the other hand, resveratrol may possess antifungal and antibacterial capabilities by impeding DNA synthesis or disrupting cell membrane potential. Additionally, resveratrol promotes wound angiogenesis by regulating the SIRT1-FOXO1-c-Myc signaling pathway. It also facilitates wound healing by modulating the miR-212/CASP8 axis to enhance cell proliferation and migration [214,215,216].

Firstly, resveratrol-loaded wound dressings facilitate wound healing through their antimicrobial, antioxidant, and inflammation-attenuating properties. Yang et al. developed a novel pH-responsive hydrogel loaded with resveratrol, where the antimicrobial and antioxidant activities increased proportionally with the resveratrol content, concurrently promoting wound healing by dampening inflammatory responses [217]. Secondly, resveratrol-loaded wound dressings also mitigate the inflammatory response by promoting macrophage polarization and modulating cytokine levels. Zhu et al. engineered a resveratrol-loaded hydrogel that alleviated the inflammatory response by inhibiting the expression of pro-inflammatory factors iNOS and TNF-α, secreted by macrophages, while enhancing the expression of anti-inflammatory factors Arg-1 and TGF-β1. This anti-inflammatory mechanism potentially involves promoting the expression of extracellular purinergic signaling pathway-associated CD73 and the adenosine 2A receptor, inducing macrophage polarization from the M1 to the M2 phenotype, suppressing the release of inflammatory cytokines and ROS production, and promoting angiogenesis, wound repair, and healing [218]. Additionally, resveratrol prevents acute inflammation in larger traumatic wounds. Tan et al. formulated a hydrogel loaded with resveratrol, where the sustained release of resveratrol heightens the secretion of anti-inflammatory cytokines, consistently mitigating the inflammatory response and fostering fibroblast migration and collagen deposition, thus facilitating the healing of hemorrhagic wounds [219].

In summary, incorporating plant extracts into various types of wound dressings enhances wound healing through multiple mechanisms. These include antimicrobial activity, the promotion of autophagy, the modulation of macrophage polarization, the reduction of pro-inflammatory cytokine secretion, the scavenging of excess ROS to mitigate oxidative stress, the secretion of growth factors, the facilitation of collagen deposition, the promotion of cell proliferation and migration, and the stimulation of angiogenesis. However, the low water solubility of plant extracts often restricts their bioavailability, a challenge that can be overcome by encapsulating them into lipids, nanoparticles, or metal frameworks [205,220]. Tang et al. developed resveratrol nanoparticles, and Feng et al. formulated a hydrogel loaded with resveratrol nanoparticles. These strategies have demonstrated the enhanced bioavailability of plant extracts within dressings, thereby reducing inflammation through antibacterial activity, ROS scavenging, the inhibition of inflammatory signaling pathways, and cytokine modulation to effectively promote wound healing [221,222].

## 6. New Research Developments and Perspectives

Immunomodulation is pivotal in wound healing across its inflammatory, proliferative, and regenerative phases, prompting an increased focus on designing dressings to enhance wound healing through this mechanism. Recent research has explored promising avenues, such as mitochondrial grafting materials and manipulations of biomaterial morphology (including structure, surface micropatterns, fiber arrangement, diameter, porosity, pore size, particle diameter, etc.) to influence immunomodulation. Additionally, advancements include integrating metal ions into dressings, combining hydrogels with microneedles or nanofibrous membranes in composites, and utilizing electrostimulation dressings with electrical conductivity. These biomaterials exhibit immunomodulatory capabilities that facilitate wound healing by regulating oxidative stress, modulating macrophage behavior, dampening inflammatory responses, and promoting tissue regeneration. However, there remains a scarcity of comprehensive studies on these materials, alongside several challenges such as unclear immunomodulatory mechanisms and issues in ensuring safety. Addressing these gaps requires further in-depth exploration by researchers in the field.

### 6.1. Mitochondrial Transfer Material and Wound Immunomodulation

Mitochondrial transfer involves restoring mitochondrial dysfunction by replacing abnormal mitochondria within a cell with healthy ones obtained from platelets, skeletal muscle, or mesenchymal stem cells, with platelets being the primary source [137,223]. These mitochondria, sourced from megakaryocytes in the bone marrow, harbor functional properties crucial for inflammation and wound healing [224]. Mitochondrial translocation modulates the inflammatory response and facilitates wound closure, particularly evident during the early stages of wound healing [137,225]. Notably, mitochondrial transfer offers significant advantages. Firstly, autologous mitochondrial transfer avoids triggering an immune response. Secondly, it maintains the extracellular microenvironment’s integrity, mitigating potential safety concerns such as biocompatibility and cytotoxicity. Furthermore, the process of isolating and transferring mitochondria is simple, efficient, easily controllable, and stable [226].

Harnessing immunomodulation via the incorporation of mitochondria into biomaterials, such as hydrogels, to bolster wound healing represents a promising avenue of research. Parisa Hassanpour et al. combined mitochondria isolated from mesenchymal stem cells with alginate/gelatin hydrogel to develop a mitochondria-transferring hydrogel [227]. This hydrogel enhanced in situ ATP production in hypoxic cardiomyocytes through mitochondrial transplantation, reduced ROS accumulation, and accelerated vascular regeneration, thereby promoting angiogenesis in rats with acute myocardial infarction. ATP provision supports metabolism, while ROS scavenging attenuates the inflammatory response during wound healing, underscoring the potential of designing wound dressings that facilitate mitochondrial transfer to enhance wound healing. Hydrogels capable of delivering mitochondria can be engineered by encapsulating mitochondria in hydrogel particles to mimic artificial cells or by mixing a mitochondria isolation buffer solution with HA-MC hydrogel, followed by repeated mixing, centrifugation, and refrigeration cycles [228,229]. To date, no dressing has been specifically engineered to promote wound healing through mitochondrial transfer, highlighting a promising area for future exploration. Importantly, unshielded isolated mitochondria encounter survival challenges when directly transplanted. In contrast, stem cells loaded with external mitochondria can employ a Trojan Horse-like delivery approach to sustain mitochondrial activity. This concept offers valuable inspiration for the future design of mitochondria-transfer wound dressings [230].

### 6.2. Biomaterial Morphology Influences the Immunomodulatory Mechanisms of Wound Healing

When a biomaterial is applied to cover a wound, it initiates an interaction with the host immune defense system, triggering a foreign body response and influencing the immunomodulation of wound healing [231]. As emphasized in the review by Dhivya Venugopal et al., the morphology of electrostatically spun nanofiber meshes—encompassing factors like fiber orientation, diameter, and porosity—crucially influences cellular immune response and repair mechanisms. For example, thicker fiber diameters are associated with increased presence of M2 macrophages. Moreover, specific mesh structures and aligned fiber orientations enhance wound healing by promoting the recruitment of macrophages and T cells, and by facilitating macrophage polarization [232]. Furthermore, the morphology of other biomaterial types profoundly influences immune response during wound healing. This includes surface micropatterns, particle diameter, material structure, porosity, pore size, etc., all of which can impact skin and immune cells, directing differential immune responses. This is mainly reflected in the following aspects.

Firstly, the surface morphology of a dressing can influence macrophage behavior. N. O. Monteiro et al. established four polycaprolactone membranes with varied surface morphologies. When macrophages were cultured on monolayers of *Escherichia coli* and *Staphylococcus epidermidis* polycaprolactone membranes with diverse surface morphologies, macrophages exhibited an M1-like phenotype; conversely, when cultured on eggshell membranes and L92 monolayer cell membranes with distinct surface morphologies, macrophages displayed an M2-like phenotype [233]. This suggests that modifying the morphology of wound dressings can influence the macrophage phenotype, thereby regulating inflammation and facilitating wound healing. Additionally, the particle diameter within injectable hydrogels also plays a crucial role in their immunomodulatory effects during wound healing. For instance, Liu et al. investigated Microporous Annealed Particle (MAP) scaffolds composed of spherical microgels with diameters of 40 μm, 70 μm, and 130 μm. Their findings revealed that MAP scaffolds featuring 130-μm diameter microgels could rebalance pro-regenerative macrophage responses, reduce inflammation, and enhance collagen regeneration, ultimately promoting more effective wound healing [234]. Thirdly, the pore size of biomaterials can also influence the immune response in wound healing. Furthermore, due to their highly tunable properties, 3D printing materials offer potential for modulating immune responses in wound healing by customizing pore size, porosity, and morphology. Cui et al. utilized 3D printing technology to customize chitosan/glycerol bioink with three micropatterns (strips, sheets, and grids) printed on commercial dressings. All three micropatterns exhibited potent antimicrobial properties, with strip micropatterns displaying the highest cellular activity and lowest haemolysis compared to the other two, and could mitigate the inflammatory response by inhibiting TNF-α and IL-1β, promoting collagen deposition, and providing a conducive microenvironment for wound healing [235]. Additionally, 3D-printed materials can modulate immune response by adjusting material porosity. Li et al. fabricated three different pore sizes (P200, P400, and P600) of polycaprolactone/polyethylene glycol/hydroxyapatite scaffolds via 3D printing. Among these, P600 exhibited the strongest ability to induce M2 macrophages, attenuate foreign body reaction, and promote angiogenesis and neointegration [236]. Unfortunately, dressings tailored to modulate immune response through a customized morphology via 3D printing technology specifically for promoting skin wound healing have not been developed to date, underscoring the need for future research in this area. Additionally, the mechanism by which dressing morphology influences immune regulation in wound healing and inflammatory response requires further exploration to elucidate in detail.

### 6.3. Immunomodulatory Role of Metal Ions in Skin Wound Repair

Metal ions offer an alternative to antibiotics, circumventing issues of resistance and long-term adverse reactions [237]. They exhibit diverse immunomodulatory effects, including antibacterial, anti-inflammatory, and antioxidant properties, along with the ability to regulate macrophage behavior and modulate cytokine expression, thereby promoting cell proliferation, migration, collagen deposition, angiogenesis, and tissue regeneration, all of which are vital for wound healing.

Metal ions primarily regulate immunity in wound healing through two key mechanisms. Firstly, they mitigate oxidative stress by clearing ROS, thus protecting cells from damage and fostering wound healing. For instance, Zhang et al. developed a hydrogel loaded with zinc oxide nanoparticles, where the released Zn^2+^ inhibited oxidative stress, cleared ROS and nitrogen, induced M2 macrophage polarization, reduced inflammation, exhibited antibacterial effects against MRSA, and promoted collagen deposition and neovascularization through the PI3K/AKT pathway, thereby facilitating MRSA-infected wound healing [238]. Similarly, Wang et al. devised a microneedle patch loaded with MgH_2_, which reduced inflammation by promoting M2 macrophage polarization and ROS clearance, concurrently fostering tissue regeneration and diabetic wound healing [239]. Additionally, UnJin Ryu et al. engineered a hydrogel loaded with a zirconium organic framework, which curbed chronic inflammatory responses by eliminating pro-inflammatory mediators like ROS, NO, and pro-inflammatory cytokines, while also enhancing cell activation, proliferation, and wound healing [240]. Secondly, metal ions exhibit potent antimicrobial properties and modulate wound healing by influencing macrophage polarization and cytokine levels. For example, Liu et al. fabricated a magnesium-doped nanofibrous membrane with robust antibacterial activity against Gram-positive/negative strains, which modulated macrophage inflammatory responses, upregulated anti-inflammatory factors, downregulated pro-inflammatory factors, and promoted angiogenesis to foster wound healing [241]. Ge et al. synthesized nanoparticles composed of a Co-based metal–organic framework, wherein Co^2+^ generated exogenous ROS to deactivate bacteria and disrupt biofilms. This activation of the immune system at the wound site promoted M1 to M2 macrophage polarization, T-cell activation, and dendritic cell maturation, thereby enhancing immune activity and facilitating wound healing [242]. Similarly, Guo et al. developed a self-assembled hydrogel of bola molecules loaded with Cu^2+^, augmenting the hydrogel’s antimicrobial capacity. Cu^2+^ upregulated CD31 and alpha-smooth muscle actin expression, mitigated the pro-inflammatory factor IL-6, thereby reducing inflammation and promoting angiogenesis, collagen deposition, and wound epithelial formation to expedite diabetic wound healing [243]. Furthermore, metal ions can enhance hydrogel mechanical properties without additional cross-linking agents, averting the biosafety and biocompatibility concerns associated with such agents. Li et al. engineered an iron ion-loaded hydrogel, endowing it with self-repairing ability, tissue adhesion, and shape adaptability. Moreover, the iron ions conferred antimicrobial, anti-inflammatory (by promoting anti-inflammatory macrophage polarization), and antioxidant properties, thereby promoting diabetic wound healing [244].

Secondly, incorporating multiple metal ions into materials simultaneously can provide various efficacies to meet diverse requirements across different stages of wound healing. Huo et al. developed a hydrogel loaded with Cu^2+^ and Zn^2+^, releasing Cu^2+^ initially to induce a pro-inflammatory response for bacterial sterilization, followed by Zn^2+^ release to inhibit the MAPK signaling pathway, thereby promoting macrophage phenotypic transition from M1 to M2 and creating an anti-inflammatory microenvironment conducive to wound healing [245]. Moreover, multiple metal ions may exhibit catalytic effects; for instance, Zn^2+^ can enhance the antibacterial efficacy of Cu^2+^. Luo et al. designed a nanofiber dressing loaded with Zn^2+^ and Cu^2+^, where Zn^2+^ acted as an immunotherapeutic “facilitator,” triggering the release of Cu^2+^ and HS from CuS for anti-inflammatory effects and promoting M2 macrophage polarization to facilitate wound healing [246]. Tian et al. developed a hydrogel wound dressing loaded with a Fe-Cu bimetallic organic framework, which exhibited enhanced catalytic activity with a 5-fold increase after Cu^2+^ doping. This cascade catalytic system demonstrated excellent antimicrobial and anti-inflammatory properties, inducing M2 macrophage polarization, remodeling the wound’s immune microenvironment, and promoting nerve and blood vessel regeneration for wound healing [247].

However, challenges remain in the use of metal ions. Controlling the concentration of metal ions is difficult, with excessive amounts potentially leading to cytotoxicity, while insufficient amounts may compromise their efficacy in promoting wound healing [248]. Additionally, the molecular mechanisms underlying the involvement of metal ions in immunomodulation for wound healing have not been fully elucidated, necessitating further research in this area.

### 6.4. Immunomodulation in Wound Healing by Conductive Electrical Stimulation Dressings

Electrostimulation demonstrates potent antimicrobial and antioxidant activity, promoting macrophage migration and polarization towards the M2 phenotype while reducing inflammatory responses. Simultaneously, it enhances cell migration, proliferation, collagen deposition, angiogenesis, and re-epithelialization, thereby fostering wound healing throughout all stages. Dressings incorporating electroactive substances such as conductive polymers, carbon nanomaterials, and metal-based materials—found in hydrogels, nanofiber membranes, collagen membranes, and foams—impart conductive properties. These dressings exhibit antibacterial, anti-inflammatory, antioxidant, and tissue regeneration effects, leveraging endogenous and wound closure currents induced by electrical stimulation. Importantly, electrical stimulation represents a physically based stimulus characterized by high biosafety and the absence of drug resistance [3,249,250].

Firstly, dressings endowed with conductive abilities can be electrically stimulated to deliver broad-spectrum antibacterial effects without inducing resistance. Song et al. developed an electroactive nanocomposite membrane with conductive properties. The dressing’s surface charge increases intracellular ROS levels in bacteria, causing oxidative damage to bacterial membranes, reducing bacterial activity, preventing biofilm formation, and imparting excellent broad-spectrum antimicrobial effects against both Gram-positive and Gram-negative bacteria [32]. Secondly, electrically stimulated hydrogels can mitigate the inflammatory response and promote wound healing. Wang et al. engineered a self-powered piezoelectric composite hydrogel capable of converting mechanical energy generated by rat activity into electrical energy. This hydrogel delivers real-time uniform and symmetrical piezoelectric stimulation to wounds, thereby modulating macrophage polarization from the M1 phenotype to the M2 phenotype, downregulating inflammatory factor levels, activating the AKT and ERK1/2 signaling pathways, and enhancing the secretion of growth factors, collagen deposition, angiogenesis, and re-epithelialization, thus facilitating wound healing [33]. Similarly, Li et al. developed a microneedle incorporating a self-powered friction nanogenerator. By releasing exogenous electric currents, it inhibits the expression of pro-inflammatory factors such as TNF-α and IL-6, attenuating the inflammatory response. Additionally, it inactivates *Staphylococcus aureus* and *Escherichia coli*, promotes tissue regeneration, and accelerates wound healing [119]. Furthermore, electrical stimulation can synergize with metal ions, resulting in enhanced wound healing effects. Zhang et al. fabricated a wearable ionic friction nanogenerator patch that converts mechanical energy from human movement into electrical energy. This patch continuously releases metal ions (Cu^2+^/Fe^2+^/Ca^2+^), exerting a “cocktail effect” on wounds, which promotes collagen deposition and angiogenesis while alleviating inflammation. This synergistic approach effectively facilitates diabetic wound healing by reducing inflammation and promoting collagen deposition and angiogenesis [251].

Although a number of conductive dressings loaded with drugs or metal ions can promote wound healing through immunomodulation and reduce inflammation, most studies have examined the anti-inflammatory and immunomodulatory effects of the loaded drugs or metal ions themselves, and few studies have investigated whether electrical stimulation alone can provide anti-inflammatory and immunomodulatory effects, and whether the use of electrical stimulation alone without the loading of drugs and metal ions can avoid biotoxicity, cytotoxicity, biosafety, and drug resistance. The use of electrical stimulation without the loading of drugs and metal ions can avoid biotoxicity, cytotoxicity, biosafety, and drug resistance, and reduce the difficulty and cost of dressing production.

### 6.5. Composite Materials

In recent years, there has been a notable shift towards developing composite wound dressings that integrate various materials and technologies. These composites offer several advantages by combining the strengths of different materials, thereby addressing individual limitations such as inadequate skin adhesion, poor biocompatibility, low mechanical properties, and non-biodegradability. By enhancing both the physical and chemical properties of dressings and expanding the range of available materials and therapeutic agents, these composites show promise in advancing wound care. Moreover, their multifaceted composition enhances their immunomodulatory capabilities, further boosting their potential for promoting efficient wound healing. Integration with 3D printing technology can further enhance precision, reproducibility, and the customization of these dressings, heralding significant advancements in the field of wound care.

#### 6.5.1. Hydrogel-Microneedle Composites

Hydrogels can provide a moist environment for microneedles and enhance their anti-inflammatory efficacy. Ning et al. developed a hydrogel microneedle with a bilayer structure loaded with Mg^2+^ ions. The moisture provided by the hydrogel bolstered the interaction between magnesium ions and the inflammatory microenvironment. Additionally, the hydrogel bolstered the mechanical properties, biocompatibility, and skin adherence of the microneedle dressings [252]. Likewise, Guo et al. developed a hydrogel microneedle dressing loaded with insulin. The hydrogel material improved the mechanical properties, biocompatibility, and skin adhesion of the microneedle dressing. It also facilitated insulin release, reduced inflammatory infiltration, mitigated the inflammatory response of the wound, promoted collagen deposition and tissue regeneration, and enhanced glycemic control and wound healing [253].

#### 6.5.2. Hydrogel Composites with Nanofibrous Membranes

The amalgamation of hydrogels and nanofibrous membranes can leverage the advantages of both while mitigating their respective drawbacks. One approach to amalgamating these materials involves designing multilayer structures, enabling the utilization of materials with inferior mechanical properties for wound dressings. Sun et al. devised a four-layer composite wound dressing by combining electrostatically spun nanofibrous membranes with hydrogels. This composite comprises a bioactive hydrogel film, a large pore size fibrous membrane, a drug-carrying hydrogel, and a small pore size fibrous membrane. The bioactive hydrogel film mimics the ECM, providing a scaffold for cell differentiation, proliferation, and collagen synthesis. The large pore-sized fibrous layer shields the bioactive layer from deformation and facilitates the transport of drugs from the drug-carrying hydrogel layer. The drug-carrying hydrogel layer maintains a moist wound environment, absorbs exudate, releases anti-inflammatory drugs steadily, and regulates the wound microenvironment in conjunction with the bioactive layer. The dense structure of the small pore-sized fibrous membrane layer primarily prevents external infections and counteracts the low mechanical properties of the drug-carrying hydrogel after swelling, thereby inhibiting the inflammatory response of macrophages and promoting wound healing [254]. Alternatively, hydrogel can be utilized to create a nanofibrous membrane. Wu et al. developed an aligned hydrogel nanofibrous membrane dressing, harnessing the combined advantages of hydrogel and nanofibrous membrane technologies. The hydrogel mimics ECM properties with its high water content, biocompatibility, degradability, superior drug-carrying capacity, and ability to enhance cell adhesion and metabolite exchange. Concurrently, the aligned nanofiber membrane’s pore structure acts as a scaffold for cell adhesion, proliferation, and guided growth, thereby accelerating wound healing. This dressing also mitigates the inflammatory response by scavenging ROS and reducing the secretion of pro-inflammatory cytokines such as IL-6 and TNF-α. Additionally, it demonstrates potent antimicrobial properties, further enhancing its efficacy in promoting wound healing [255].

#### 6.5.3. Composite Material Combining an Aerogel and a Nanofiber Membrane

Han et al. devised a bilayer dressing comprising an aerogel and an electrostatically spun nanofibrous membrane. The outer nanofibrous membrane serves to prevent bacterial infiltration and furnish mechanical reinforcement, whereas the inner aerogel layer absorbs wound exudate and initiates H2S autocatalysis. This dual-layer dressing effectively scavenges ROS, diminishes inflammation, fosters vascular regeneration, and expedites wound healing [256].

#### 6.5.4. Composites Incorporating 3D Printing Technology

The integration of 3D printing technology with composite materials offers significant advantages due to its precision, high reproducibility, and customizability. Cao et al. introduced a novel wound dressing composed of conductive hydrogel strips and a nanofibrous membrane. This dressing was created by 3D printing hydrogel onto a nanofibrous membrane loaded with doxycycline hydrochlorid. This innovative dressing demonstrates robust antimicrobial properties by effectively scavenging ROS. It also promotes macrophage polarization towards the M2 phenotype, decreases inflammatory factor levels, mitigates the inflammatory response, stimulates endothelial cell migration, and accelerates wound healing [257]. Similarly, Zhang et al. devised an innovative 3D-printed drug-loaded hydrogel microneedle dressing. This dressing attenuates inflammatory responses, promotes vascular endothelial growth factor (VEGF) secretion, accelerates tissue repair, and enhances wound healing [258].

Nonetheless, research on composites endowed with immunomodulatory capabilities remains relatively scarce. A comprehensive exploration of their advantages, characteristics, and underlying mechanisms is warranted, emphasizing the need for further studies in this direction.

## 7. Summary

A diverse array of skin wound dressings exists, offering numerous avenues for improvement through both physical and chemical means to enhance their immunomodulatory properties. Numerous in vivo and in vitro animal studies have highlighted significant advancements in wound healing achievable by modulating the immune response. This paper emphasizes the critical role of immunomodulatory dressings and introduces several novel and effective approaches, providing a roadmap for future developments in wound dressing technology. However, current investigations into immunomodulatory wound dressings are limited by their ability to simultaneously modulate multiple aspects of the immune response. Additionally, there remains a lack of studies elucidating the roles of specific subtypes of polarized M2 macrophages, with much of the research confined to animal models. Moving forward, further exploration into these aspects is crucial, along with clinical trials to assess the therapeutic efficacy of these immunomodulatory dressings.

## Data Availability

Not applicable.

## Abbreviations

ROS	Reactive oxygen species
OxPhos	Oxidative phosphorylation
ECM	Extracellular matrix
MMPs	Matrix metalloproteinases
AgNPs	Silver nanoparticles
ILs	Interleukins
TNF-α	Tumor Necrosis Factor-α
MCP-1	Monocyte chemoattractant protein-1
AIDS	Acquired immunodeficiency syndrome
HIV	Human immunodeficiency virus
CRRT	Continuous renal replacement therapy
PVA	Poly(vinyl alcohol)
VEGF	Vascular endothelial growth factor
NO	Nitric oxide
Th2	T helper 2
MRSA	Meticillin-resistant *Staphylococcus aureus*
AS-IV	Astragaloside IV
